# Murine Aβ_4–x_ Peptides Retain High-Affinity Redox-Silent Cu^2+^ Binding

**DOI:** 10.1021/acs.inorgchem.6c04247

**Published:** 2026-10-23

**Authors:** Simon C. Drew

**Affiliations:** Division of Biophysics, Institute of Experimental Physics, Faculty of Physics, University of Warsaw, Warsaw 02-093, Poland

## Abstract

β-Amyloid peptides N-truncated at Phe4 (Aβ_4–x_) contain an amino-terminal Cu- and Ni-binding (ATCUN) motif that can form high-affinity, redox-silent Cu^2+^ complexes. Human Aβ_4–40/42_ is present in the brains of individuals with and without Alzheimer’s disease, and soluble Aβ_4–9_ can be generated by neprilysin proteolysis of Aβ_1–40/42_ in vitro. These observations led to the proposal that Aβ_4–x_ may be functional Cu^2+^-binding peptides. Because amyloid precursor protein is constitutively expressed in mammals, this study assessed whether murine Aβ_4–x_ peptides retain the properties of the human analogues. Cu^2+^ coordination, binding affinity, and redox activity were compared for murine and human Aβ_4–9_ and Aβ_4–16_. The murine peptides formed Cu^2+^ complexes with spectroscopic parameters closely matching those of the human peptides. Competitive binding against glycylglycylhistidine gave conditional Cu^2+^ dissociation constants at pH 7.4 of 3–4 fM for murine Aβ_4–x_ and 8–10 fM for human Aβ_4–x_. All Cu­(Aβ_4–x_) complexes were redox-silent in an aminophenyl fluorescein/ascorbate assay. Thus, murine Aβ_4–x_ has the chemical capacity required for a Cu^2+^-binding role if generated in vivo. However, because endogenous murine Aβ_4–x_ has not been detected in wild-type mice, the present findings challenge the support for a broadly conserved physiological role for Aβ_4–x_ in mammalian Cu^2+^ handling.

## Graphical abstract

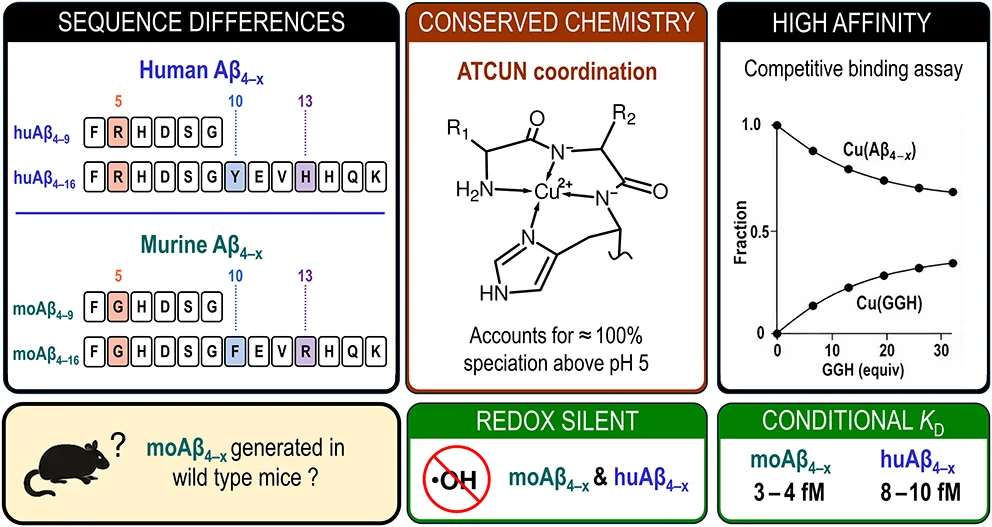

## 1 Introduction

Accumulation of β-amyloid (Aβ) species derived from sequential processing of the amyloid precursor protein (APP) by β-site APP cleaving enzyme 1 (BACE1) and γ-secretase is a pathological hallmark of Alzheimer’s disease (AD).^1,2^ Although Aβ_1–40_ and Aβ_1–42_ remain the most extensively studied Aβ species, N-terminally truncated proteoforms are also abundant components of the Aβ pool, including peptides beginning at Phe4 (Aβ_4–x_). From a bioinorganic perspective, this truncation is important because it creates a free N-terminal Xaa–Yaa–His sequence known as the amino-terminal Cu^2+^- and Ni^2+^-binding (ATCUN) motif. The Cu^2+^ coordination chemistry of ATCUN motifs has been extensively investigated in short peptides and proteins, including thermodynamic, spectroscopic, kinetic, and applied studies.^3–7^ At sufficiently high pH, this motif can form a {H_2_N, 2N^–^, N_Im_} first coordination sphere involving the free N-terminal amine, the histidine imidazole nitrogen, and two intervening deprotonated backbone amides within three fused chelate rings.

Unlike the weakly bound and redox-active Cu complexes of canonical Aβ_1–x_ peptides, human (hu) Aβ_4–x_ binds Cu^2+^ with low-femtomolar conditional dissociation constants at physiological pH,^8,9^ and Cu­(huAβ_4–x_) complexes show negligible Cu-mediated redox activity under conditions where Cu­(huAβ_1–x_) can promote reactive oxygen species (ROS) production.^8,10^ Thus, if Cu is associated with Aβ in vivo—an issue that remains contentious^11,12^ —the known coordination chemistry points to huAβ_4–x_ as the Aβ proteoform more likely to bind Cu^2+^.

Human Aβ_4–x_ peptides are therefore of interest not only as N-truncated amyloid species but also as high-affinity Cu^2+^-binding peptides. huAβ_4–x_ (*x* ≥ 40) has been detected in the brain and cerebrospinal fluid of individuals with mild cognitive impairment, sporadic AD, and familial AD, and also in the cortex of persons without diagnosed dementia.^13–20^ huAβ_4–x_ immunoreactivity has also been observed in transgenic AD mouse models expressing human APP variants and in aged dogs with cerebral vascular amyloid and parenchymal preamyloid lesions.^18,21^ Although the proteolytic origin of huAβ_4–x_ remains incompletely resolved, disintegrin and metalloproteinase with thrombospondin motifs 4 (ADAMTS4) has been shown to generate huAβ_4–x_ both from canonical huAβ_1–x_ peptides and directly from human APP through a BACE1-independent pathway.^22,23^ In addition, soluble huAβ_4–9_ is produced during *in vitro* degradation of huAβ_1–x_ by recombinant human neprilysin.^24^ Thus, the short huAβ_4–9_ fragment is of interest as a potential soluble catabolic product, whereas longer huAβ_4–x_ species are relevant to deposited or aggregation-prone Aβ pools.

The murine (mo) Aβ sequence differs from the human sequence at residues 5, 10, and 13, corresponding to R5G, Y10F, and H13R substitutions (Table 1). These differences are associated with lower production of canonical moAβ_1–x_ species from APP and a lower aggregation propensity of moAβ_1–x_ relative to huAβ_1–x_.^25,26^ The murine Aβ sequence within APP has been associated with greater β′-site APP cleavage and generation of Aβ_11–x_ species compared with the corresponding human sequence.^27^ Whether endogenous murine APP or moAβ substrates efficiently generate moAβ_4–x_ species remains unresolved.

**Table 1:** Five ATCUN Peptides Used in This Study^a^

Peptide	Sequence
huAβ_4–9_	H_2_N–F**R**HDSG–OH
moAβ_4–9_	H_2_N–F**G**HDSG–OH
huAβ_4–16_	H_2_N–F**R**HDSG**Y**EV**H**HQK-NH_2_
moAβ_4–16_	H_2_N–F**G**HDSG**F**EV**R**HQK-NH_2_
GGH	H_2_N-GGH–OH

a Boldface highlights the differences between human (hu) and murine (mo) β-amyloid (Aβ) sequences. Aβ_4–16_ peptides with a C-terminal amide were used to model Cu^2+^ binding of longer Aβ_4–x_ (x = 40, 42) species without the complication of C-terminally mediated aggregation. Aβ_4–9_ peptides with a free C-terminus were used to study Cu^2+^ binding of soluble, catabolic peptide fragments generated by neprilysin. Residue numbering follows that of the canonical Aβ_1–x_ sequence.

The biological interpretation of Aβ_4–x_ Cu^2+^ binding therefore depends on two separable questions. The first is chemical: whether Aβ_4–x_ peptides from other mammals retain the high-affinity, redox-silent Cu^2+^-binding properties of the human sequence. The second is biological: whether the corresponding peptides are generated from endogenous APP or Aβ substrates at relevant concentrations and locations. A conserved physiological role in Cu^2+^ handling would require both. Conversely, if Aβ_4–x_ is generated mainly under pathological conditions, the same high-affinity Cu^2+^ binding could instead contribute to disease-associated Cu sequestration rather than to local Cu-mediated ROS production. In either case, the essential starting point is to determine whether the intrinsic Cu^2+^ coordination chemistry is conserved among mammalian species with constitutive APP expression.

In this study, the Cu^2+^ coordination chemistry of murine Aβ_4–x_ peptides was therefore characterized using pH-dependent UV–vis and room-temperature EPR spectroscopies and a fluorescence-based assay of Cu-mediated ROS production. moAβ_4–9_ was studied as the murine analogue of the short, soluble huAβ_4–9_ fragment, while moAβ_4–16_ was used as a soluble model for C-terminally extended moAβ_4–x_ peptides. The Cu^2+^ coordination, binding affinity, and redox activity were compared with that previously described for huAβ_4–x_ to determine the influence of the R5G, Y10F and H13R substitutions in the murine sequence.

## 2 Methods

### 2.1 Reagents

Ultrapure water with resistivity of 18 MΩ cm^–1^ was used throughout. Peptides used in this study are shown in Table 1. GGH (free C-terminus) was obtained from Sigma–Aldrich (G4541, purity >98%, source BCCK0499) used without further purification. Peptides huAβ_4–9_ (free C-terminus), huAβ_4–16_ (amidated C-terminus), and moAβ_4–16_ (amidated C-terminus) were synthesized by Mimotopes (Melbourne, Australia), and moAβ_4–9_ (free C-terminus) was synthesized by ProteoGenix (Schiltigheim, France). The huAβ_1–16_ peptide (free C-terminus) was synthesized by Modpep (Melbourne, Australia). Peptide identity and purity (>95%) were confirmed using ESI-MS and reverse-phase high-performance liquid chromatography.

Stock solutions of all peptides were prepared in ultrapure water, and their concentrations were determined at 214 nm in quartz cuvettes (Hellma Analytics, 108B-10–40) using a Cary 100 Bio UV–vis spectrophotometer and the following calculated extinction coefficients:^28^ GGH, 7013 M^–1^ cm^–1^; huAβ_4–16_, 38468 M^–1^ cm^–1^; moAβ_4–16_, 33189 M^–1^ cm^–1^; huAβ_4–9_, 15155 M^–1^ cm^–1^; moAβ_4–9_, 15074 M^–1^ cm^–1^; huAβ_1–16_, 40482 M^–1^ cm^–1^.

Natural abundance ^63,65^CuCl_2_ (Sigma–Aldrich, 307483) was prepared in ultrapure water. Monoisotopic ^65^Cu was prepared by dissolving ^65^CuO (Cambridge Isotope Laboratories, CULM-4577) in concentrated H_2_SO_4_, removing excess acid under heat, and then adding ultrapure water. After measuring the absorbance at 815 and 450 nm using a SPECTROstar Nano spectrophotometer (BMG Labtech, Germany) with a 1 cm-path length cuvette (Precision Cells, 701MES10.50A), the concentrations of copper stock solutions were determined from the absorbance difference *A*_815_ – *A*_450_ and an absorptivity of 12.6 M^–1^ cm^–1^.

### 2.2 Sample Preparation

Protein LoBind tubes (Eppendorf) were used for all sample containment. For pH-metric titrations, ^65^Cu/peptide 0.8:1 solutions were prepared in 100 mM NaCl (Sigma, S3014) at a final Cu^2+^ concentration of 1 mM. The solution pH was incrementally raised from a starting pH ≈ 2 by adding small quantities of concentrated NaOH (Sigma, S8045), and the pH at each step was measured using a Biotrode combined glass electrode (Metrohm, 6.0224.100). The pH adjustment changed the sample volume throughout the titration by <1%.

For competition binding experiments, ^65^Cu/Aβ_4–x_/GGH 0.83:1:*n* (0 ≤ *n* ≤ 30) solutions were prepared in 100 mM NaCl at a final Cu^2+^ concentration of 0.5 mM. The solution pH was carefully raised and stabilized to 7.40 ± 0.03 by adding small quantities of concentrated NaOH and HCl (Chempur, 115752837) as required, which changed the overall sample volume by <1%. The solutions were then incubated at room temperature until Cu^2+^ binding equilibrium was achieved. Competition experiments were performed in 100 mM NaCl without added buffer to avoid additional Cu^2+^ binding equilibria involving buffer molecules. This approach is consistent with potentiometric studies of metal–peptide equilibria, which are also performed in simple supporting electrolyte rather than in complex biological buffers. Although no external buffer was added, the samples were not devoid of buffering capacity because the ionizable groups of Aβ_4–x_ and, more importantly, of the excess GGH present at most competition points provided intrinsic weak-acid/weak-base buffering during equilibration. Nevertheless, after manual adjustment, the pH was checked and fine-tuned, if necessary, during the incubation to ensure it remained within the above-mentioned range. To estimate the time required for ^65^Cu/Aβ_4–x_/GGH solutions to reach Cu^2+^ binding equilibrium, samples of ^65^Cu/Aβ_4–x_ 0.83:1 (1 mM Cu^2+^) and 12 mM GGH were separately prepared in 100 mM NaCl, and the pH of each sample was adjusted to pH 7.40 ± 0.03 using concentrated NaOH and HCl solutions. Equal volumes of the Cu/Aβ_4–x_ and GGH solutions were then combined to yield a final stoichiometry of Cu/Aβ_4–x_/GGH 0.83:1:10 (0.5 mM Cu^2+^), and the transfer of Cu^2+^ from Aβ_4–x_ to GGH was monitored by acquiring EPR spectra as a function of time. The pH was not periodically measured and fine-tuned during the kinetic assay; however, at the chosen stoichiometry, the pH was stable to within ±0.05 units.

### 2.3 Electronic Spectroscopy

pH-metric UV–visible absorption spectra were measured using a SPECTROstar Nano spectrophotometer and a quartz cuvette with a 1 cm path length and 100 mM NaCl as a baseline. All spectral analyses used raw data, but a smoothing function (0th-order polynomial including four nearest neighbors) was applied to displayed spectra.

### 2.4 EPR Spectroscopy

#### 2.4.1 Data Acquisition

Room-temperature continuous-wave EPR spectra were acquired using a CMS8400 X-band spectrometer (Adani, Belarus) fitted with a TE102 cavity, with solutions contained within a quartz flat cell (Wilmad, WG-808-Q). Measurements were made using the following settings: microwave frequency, 9.42 GHz; microwave attenuation, 10 dB; magnetic field sweep rate, 10 gauss s^–1^; magnetic field modulation amplitude, 8 gauss; magnetic field modulation frequency, 100 kHz; field resolution, 4096 points (down-sampled to 1024 points); receiver gain, 1000; time constant, 100 ms; averages, 20 (unless stated otherwise). All spectral analyses used raw data, but a smoothing function (second-order polynomial including four nearest neighbors) was applied to displayed spectra.

#### 2.4.2 Baseline Correction and Normalization

Baseline correction was carried out by subtracting the spectrum of 100 mM NaCl obtained using the same acquisition parameters. Each baseline-corrected first-harmonic spectrum (proportional to dχ″/dB_0_) was integrated twice to determine the area under the corresponding absorption curve (proportional to χ″), and the original spectrum was then normalized by dividing by this factor. Differences in the microwave frequency among spectra were corrected by shifting each experimental field position *B*_0_ by an amount *B*_0_(ν_eff_ /ν_0_ – 1), where ν_0_ is the frequency at which the spectrum was acquired and ν_eff_ = 9.420 GHz is the common frequency at which all spectra were analyzed and compared.

The experimental magnetic field was calibrated by measuring the spectrum of the DPPH radical (*g* = 2.0036) in DMSO and calculating the difference Δ*B* between the expected and measured resonance fields. Simulated principal *g* values were then corrected by an amount Δ*g* = −Δ*B g* ^2^β/(*hν*), assuming that the field miscalibration Δ*B* was constant across the measured field range.

#### 2.4.3 EPR Spectral Simulations

Room-temperature spectra were simulated using the static Hamiltonian

1 $$H=\beta_{e}B^{T}\cdot g\cdot S+S^{T}\cdot A\cdot I-g_{n}\beta_{n}B^{T}\cdot I$$

where **S** and **I** are the electron and ^65^Cu nuclear vector spin operators, **g** and **A** are the 3 × 3 electron Zeeman and ^65^Cu electron–nuclear hyperfine coupling matrices, β_e_ is the Bohr magneton, β_n_ is the nuclear magneton, and **B** is the applied magnetic field. The hyperfine interaction is proportional to the *g* factor of the relevant nucleus; thus **A**(^65^Cu) = **A**(^63^Cu) × *g*_n_(^65^Cu)/*g*_n_(^63^Cu) = 1.07 **A**(^63^Cu). Axial symmetry for **g** and **A** was assumed, with principal values of *g*_⊥_, *g*_||_ and *A*_⊥_, *A*_||_, respectively. In conjunction with the above Hamiltonian, the “chili” function in EasySpin^29^ v.6.0.6 was used to simulate room-temperature EPR spectra of Cu­(Aβ_4–x_) complexes in the slow-motion regime by solving the stochastic Liouville equation for the full diffusion tensor **D** and computing line positions to first order; and for aquated Cu^2+^ and Cu­(GGH), the “garlic” function was used to simulate the room-temperature EPR spectra in the fast-motion regime. In all cases, the diffusion tensor was assumed to be isotropic (**D** = *D*_iso_), yielding an isotropic correlation time τ_c_ = 1/(6*D*_iso_). The static (*g*_⊥_, *g*_||_, *A*_⊥_, *A*_||_, Gaussian line width) and dynamic (τ_c_) parameters were varied iteratively using the “esfit” module in EasySpin to achieve least-squares minimization of the difference between the experimental and simulated spectra.

#### 2.4.4 Decomposition of EPR Spectra of Cu/Aβ_4–x_/GGH 0.83:1:*n*

The normalized spectra of Cu/GGH 0.83:1 and Cu/Aβ 0.83:1 provided the spectra of Cu­(GGH) and Cu­(Aβ). To determine the relative proportions of Cu­(Aβ) and Cu­(GGH) in samples of Cu/Aβ/GGH 0.83:1:*n* (*n* = 1–30), a weighted summation of the CuA spectrum (*S*_A_) and CuB spectrum (*S*_B_) was made

2 $$S=f\;S_{A}+\left(1-f\right)S_{B}$$

and the weighting *f* was iteratively varied using a Generalized Reduced Gradient Nonlinear solver (Frontline Systems, Inc., www.solver.com) to minimize the mean-squared deviation between the reconstructed spectrum *S* and the experimental spectrum, the scaling of which was also permitted to be varied by ±5% to allow for normalization errors.

For each Aβ_4–x_ peptide, the entire procedure was repeated using sample sets prepared from scratch on two independent occasions. The two resulting EPR data sets were then combined and used to determine the conditional Cu^2+^ binding constant of Aβ_4–x_.

### 2.5 Determination of Conditional Cu^2+^ Binding Constants at pH 7.4

Setting A = Aβ and B = GGH, the total concentrations in a Cu/A/B mixture are:

3 $$\begin{array}{ll} {[A]}_{T} & =\;\left[A^{\prime }\right]+\left[{\left(\mathrm{CuA}\right)}^{\prime }\right]\\ {[B]}_{T} & =\;\left[B^{\prime }\right]+\left[{\left(\mathrm{CuB}\right)}^{\prime }\right]\\ {[\mathrm{Cu}]}_{T} & =\;\left[{\mathrm{Cu}}^{\prime }\right]+\left[{\left(\mathrm{CuA}\right)}^{\prime }\right]+\left[{\left(\mathrm{CuB}\right)}^{\prime }\right] \end{array}$$

where

4 $$\begin{array}{ll} \left[A^{\prime }\right] & =\;\underset{i}{\sum }[H_{i}A]\\ \left[B^{\prime }\right] & =\;\underset{i}{\sum }[H_{i}B]\\ \left[{\left(\mathrm{CuA}\right)}^{\prime }\right] & =\;\underset{i}{\sum }[{\mathrm{CuH}}_{i}A]\\ \left[{\left(\mathrm{CuB}\right)}^{\prime }\right] & =\;\underset{i}{\sum }[{\mathrm{CuH}}_{i}B]\\ \left[{\mathrm{Cu}}^{\prime }\right] & =\;\left[{\mathrm{Cu}}^{2+}\right]+\left[{\mathrm{CuOH}}^{+}\right]+\left[\mathrm{Cu}{\left(\mathrm{OH}\right)}_{2}\right]+\left[{\mathrm{CuCl}}^{+}\right]+\left[{\mathrm{CuCl}}_{2}\right] \end{array}$$

Primed quantities denote conditional concentration pools at a fixed pH. Thus, [A′] and [B′] are the total unbound ligand pools, summed over all protonation states; [(CuA)′] and [(CuB)′] are the corresponding total 1:1 Cu^2+^-bound pools, summed over protonated/deprotonated complex forms; and [Cu′] denotes Cu^2+^ not bound to A or B, including aquated, hydrolyzed and chloride-bound Cu^2+^ species.

The above equations exclude ternary CuH_*i*_AB complexes as well as binary and ternary complexes with buffer molecules. Although it is possible to formally include such species,^33^ a mixture of ATCUN peptides A and B forms only binary CuA and CuB chelates at pH 7.4, and the use of buffer-free 100 mM NaCl as the solvent in the present work further excludes competitive binding by buffer molecules.

Assuming that [Cu′] ≪ [(CuA)′] + [(CuB)′] and that all peptide-bound Cu^2+^ is detected in EPR spectra of a Cu/A/B mixture, we have

5 $$\begin{array}{l} \left[{\left(\mathrm{CuA}\right)}^{\prime }\right]=f{[\mathrm{Cu}]}_{T}\\ \left[{\left(\mathrm{CuB}\right)}^{\prime }\right]=\left(1-f\right){[\mathrm{Cu}]}_{T} \end{array}$$

where the proportion *f* is determined from the spectral decomposition described in Section 2.4.4. The conditional Cu^2+^ binding constants are defined as

$$\begin{array}{l} K_{\mathrm{CuA}}^{\prime }=\frac{\left[{\left(\mathrm{CuA}\right)}^{\prime }\right]}{\left[{\mathrm{Cu}}^{\prime }\right]\left[A^{\prime }\right]}\\ K_{\mathrm{CuB}}^{\prime }=\frac{\left[{\left(\mathrm{CuB}\right)}^{\prime }\right]}{\left[{\mathrm{Cu}}^{\prime }\right]\left[B^{\prime }\right]} \end{array}$$ 6

Because A and B compete for the same pool of free Cu^2+^ in each sample, the free copper concentration is common to both equilibrium expressions. Taking the ratio of the two expressions in eq 6 and substituting the concentration relationsin eqs 3 and 5 gives a ratio

$$R=\frac{K_{\mathrm{CuA}}^{\prime }}{K_{\mathrm{Cu}B}^{\prime }}=\frac{f\left({[B]}_{T}-(1-f{)[\mathrm{Cu}]}_{T}\right)}{\left(1-f\right)\left({[A]}_{T}-f{[\mathrm{Cu}]}_{T}\right)}$$ 7

Rearranging and collecting terms in *f* gives a quadratic equation

$$\left(R-1\right){[\mathrm{Cu}]}_{T}f^{2}+\left(-R\left({[A]}_{T}+{[\mathrm{Cu}]}_{T}\right)-{[B]}_{T}+{[\mathrm{Cu}]}_{T}\right)f+R{[A]}_{T}=0$$ 8

For each value of [B]_T_ = *n* [A]_T_ (*n =* 0–30), the physically meaningful root of eq 8 (constrained to 0 ≤ *f* ≤ 1) gives the fraction of Cu^2+^ present in the conditional pool (CuA)′ at fixed pH.

The decomposed experimental values of *f* = [(CuA)′]/[Cu]_T_ from EPR analyses of two independent sample preparations at pH 7.4 were fitted directly using least-squares nonlinear regression in GraphPad Prism version 11.02 for Windows (GraphPad Software, San Diego, California USA). The independent variable was X = [B]_T_ */* [A]_T_, and the fitted parameter was *L* = log *R*. During fitting, *R* was calculated as *R* = 10^*L*^, while [A]_T_ = 0.6 mM and [Cu]_T_ = 0.5 mM were held fixed.

After fitting the value of log *R*, the known conditional Cu^2+^ binding constant of B = GGH at pH 7.4 was then used to calculate the conditional binding constant of A = Aβ_4–x_ according to

$$\log\,K_{\mathrm{CuA}}^{\prime }=\log R+\log K_{\mathrm{CuB}}^{\prime }$$ 9

where log $K_{\mathrm{CuB}}^{\prime }$ = 12.216 ± 0.007 is the conditional binding constant calculated at pH 7.4 from the tabulated potentiometric constants for GGH in 0.1 M KNO_3_.^30^ The uncertainty in log $K_{\mathrm{CuB}}^{\prime }$ reflects the formal errors propagated from the tabulated potentiometric constants, assuming independence and ignoring covariance.

Assuming independent uncertainties, the uncertainty in log $K_{\mathrm{CuA}}^{\prime }$ was then estimated by propagating the uncertainties in the fitted log *R* value and the reference log $K_{\mathrm{CuB}}^{\prime }$ value:

10 $$\Delta \left(\log K_{\mathrm{CuA}}^{\prime }\right)\approx \sqrt{{[\Delta \left(\log R\right)]}^{2}+{[\Delta \left(\log K_{\mathrm{CuB}}^{\prime }\right)]}^{2}}$$

with $\Delta \left(\log K_{\mathrm{CuB}}^{\prime }\right)$ = 0.007 and Δ­(log *R*) being taken as half the 95% confidence interval in the fitted value of log *R*.

### 2.6 Measurement of ROS Production

Free radical production of Aβ peptides was examined at room temperature in the presence of Cu^2+^ and ascorbic acid using the fluorescent probe 2-[6-(4′-amino)­phenoxy-3H-xanthen-3-one-9-y]­benzoic acid, otherwise known as aminophenyl fluorescein (APF), which is nonfluorescent in the presence of superoxide or hydrogen peroxide but is cleaved to fluorescein upon reaction with hydroxyl radicals.^31^ The APF was purchased as a methyl acetate solution (Cayman Chemical, 10157); the solvent was evaporated and the residue dissolved at a concentration of 5 mM in dimethylformamide prior to use. Ascorbate (AscH^–^; Sigma, A5960) was prepared in ultrapure water immediately before the assay. Stock solutions of CuCl_2_ and HEPES (Sigma, H3725) pH 7.4 were prepared in ultrapure water.

To duplicate wells of a black, 96-well plate (Greiner, 675096), reagents were combined to yield a final volume of 100 μL and final concentration of 8 μM CuCl_2_, 10 μM peptide, 300 μM AscH^–^, 100 μM APF, and 20 mM HEPES pH 7.4. Solutions of CuCl_2_, peptide, and APF were premixed before adding AscH^–^ to trigger the start of the assay. Prior to the measurement at each time point, the plate was shaken for 10 s at 150 rpm in a 4 mm double orbital pattern. The fluorescence of the duplicate wells was measured at 3 min intervals at 25 °C using a POLARstar microplate reader (BMG Labtech, Germany) with 485 nm excitation and 520 nm emission filters. To prevent evaporation, the plate was covered with clear polyolefin adhesive (Nunc). The rate of ROS production was calculated from the approximately linear slope of the fluorescence growth curve in the 21–39 min range post addition of AscH^–^. The mean and standard deviation of the ROS rate from *n* = 3 independent experiments prepared and performed on different days were analyzed using GraphPad Prism.

## 3 Results and Discussion

### 3.1 UV–vis and EPR Spectra of Cu­(moAβ_4–x_) Complexes

The pH-metric UV–vis and room-temperature EPR titrations of Cu/moAβ_4–x_ 0.8:1 (Figure 1) showed that a characteristic {H_2_N, 2N^–^, N_Im_} (ATCUN) coordination environment (Figure 2B) appears at mildly acidic pH, with apparent p*K* values of 4.17 (moAβ_4–9_) and 4.27 (moAβ_4–16_), which is comparable with the pH-dependent ATCUN coordination of huAβ_4–16_ and huAβ_4–9_.^8,9^ At low pH, a {6H_2_O} (aquated Cu^2+^) coordination mode dominates, together with a minor species having optical parameters (λ_max_ ≈ 725 nm (*ε*_725_ ≈ 40 M^–1^ cm^–1^) consistent with a mixed N/O complex with a weak ligand field (Table S1, Figures S1–S2, Section S1, Supporting Information). A similar species has been identified at mildly acidic pH in Cu/GGH^30^ and other Cu/ATCUN^32^ systems (see Supplementary Discussion) and assigned to a {H_2_N, N_Im_, 2O}-type Cu^2+^ coordination mode involving the N-terminal amine, imidazole nitrogen, and two O donors/waters. Such a species was not included in the published potentiometric models of human Cu/Aβ_4–x_ complexes.^8,9^ Reinspection of the published Cu/huAβ_4–x_ data suggest that the corresponding room-temperature population, if present, is small, consistent with only minor deviations from an isosbestic point between the absorption lines of {6H_2_O} and {H_2_N, 2N^–^, N_Im_}, whereas a related component was more clearly identified in frozen-solution EPR data.^9^ Thus, the putative {H_2_N, N_Im_, 2O}-type species appears to be a genuine but minor component of Cu^2+^ speciation by Aβ_4–x_ at mildly acidic pH, with its apparent population depending on temperature and peptide sequence, which alter the relative stability of the {H_2_N, N_Im_, 2O} and {H_2_N, 2N^–^, N_Im_} coordination modes. Although the assignment of a {H_2_N, N_Im_, 2O}-type coordination mode to the intermediate-pH, mixed N/O complex is putative, its speciation is negligible above pH 5. Consequently, the remaining characterization focuses on the unambiguous {H_2_N, 2N^–^, N_Im_} coordination of Cu-bound moAβ_4–x_ at pH 7.4.

**Figure 1:**
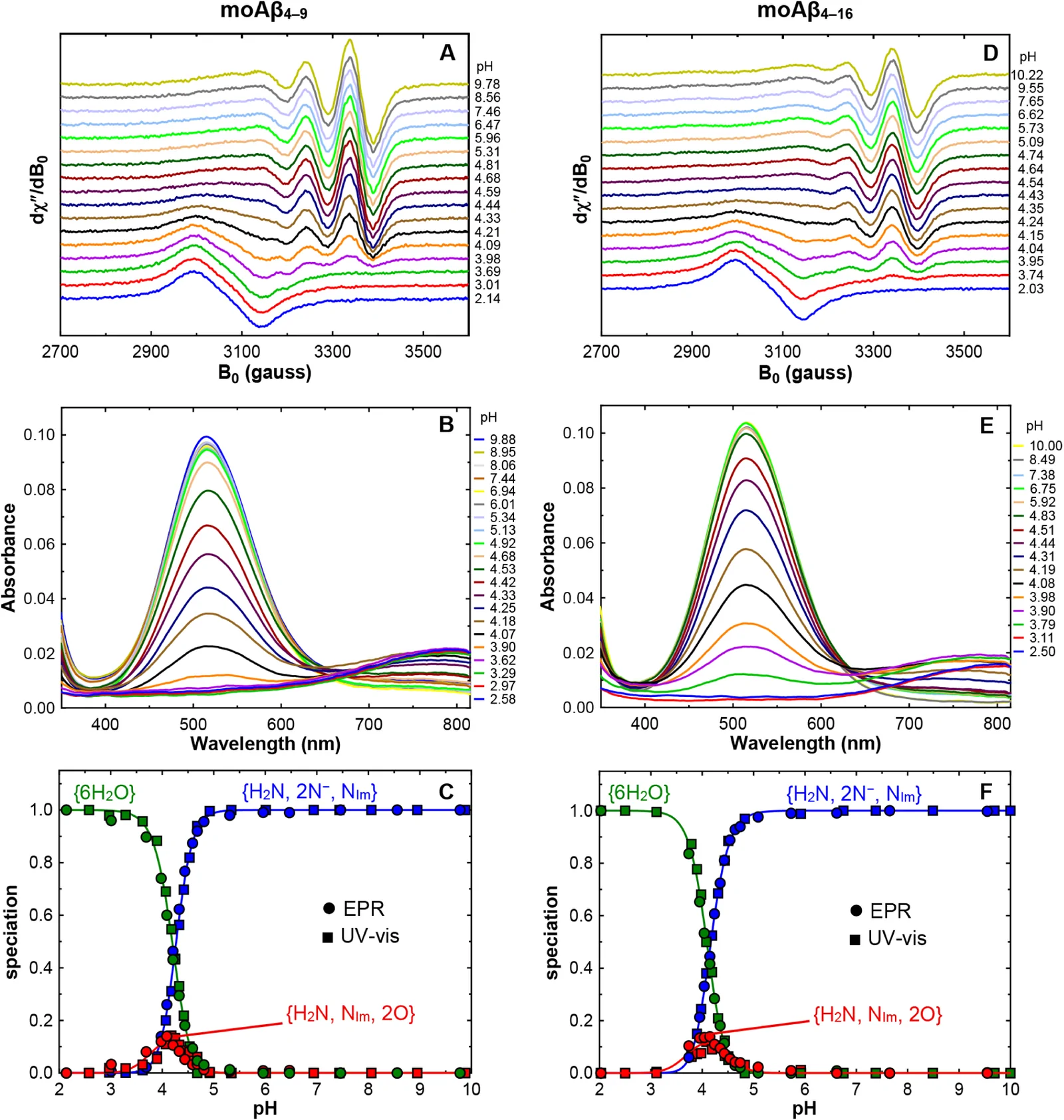
pH-metric EPR and UV–vis spectra of ^65^Cu/Aβ_4–x_ 0.8:1 in 100 mM NaCl, 25 °C ([Cu]_T_ = 1 mM). **(A–C)** moAβ_4–9_, **(D–F)** moAβ_4–16_. The Cu speciation in panels *C* and *F* was determined by decomposition of the spectra in panels *A*–*B* and *D*–*E*, respectively. Basis spectra used for the decompositions were isolated as described in the Supplementary Methods and are shown in Figure S1 (moAβ_4–9_) and Figure S2 (moAβ_4–16_). The vertical scales in panels *A* and *D* are the same.

**Figure 2:**
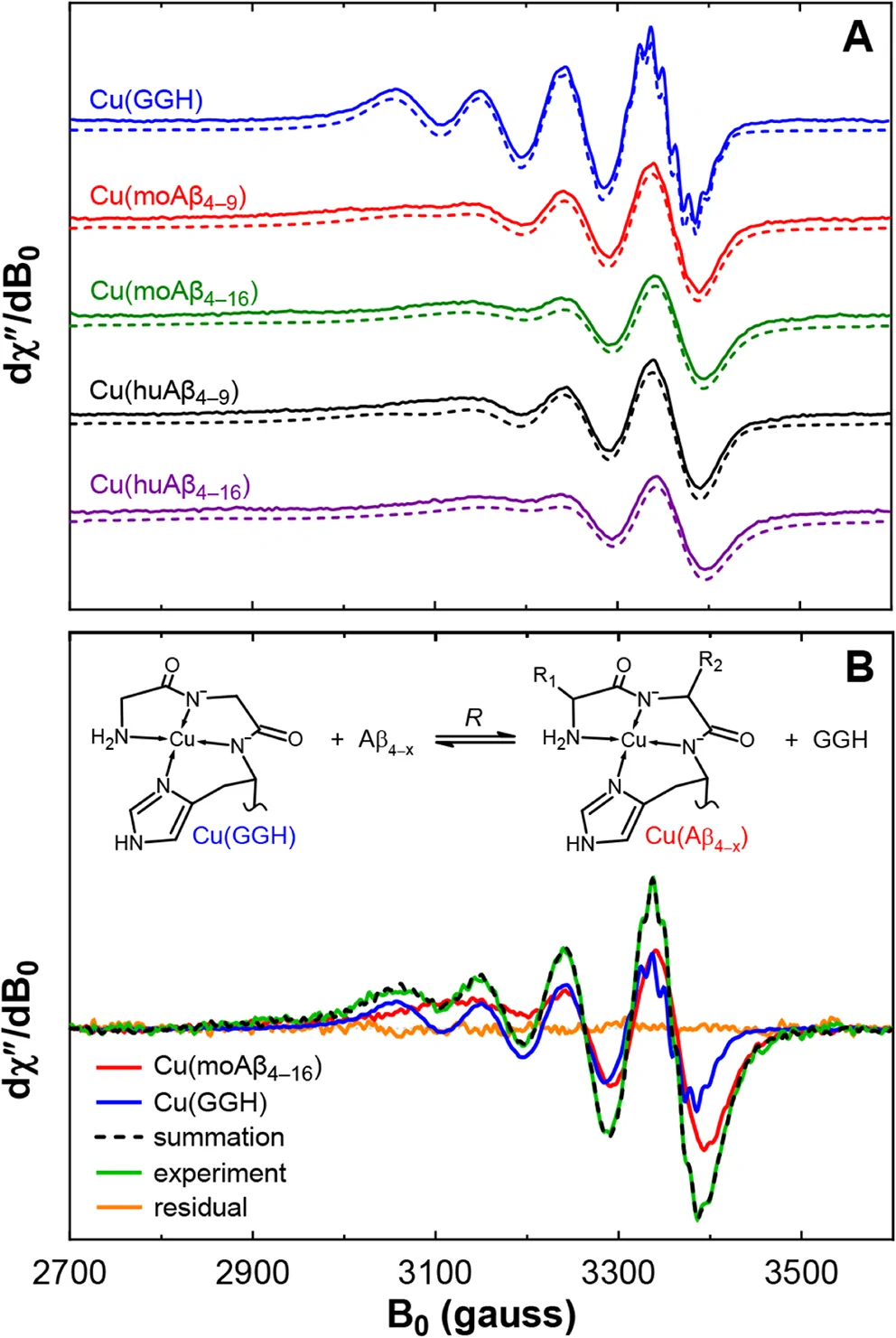
(A) Experimental (solid lines) and simulated (dashed lines) room-temperature EPR spectra of the {H_2_N, 2N^–^, N_Im_} (ATCUN) Cu^2+^ coordination modes of GGH and Aβ_4–x_ peptides with murine­(mo) and human (hu) sequences (0.5 mM ^65^Cu, 0.6 mM peptide, 100 mM NaCl pH 7.4). Experimental spectra are the average of 80 scans and normalized by their double-integrated intensity. Simulations used the parameters in Table 2. Spectra are vertically offset for clarity. (B) Representative decomposition of the spectrum of Cu/moAβ_4–16_/GGH 0.83:1:30 at pH 7.4. The reconstructed spectrum was obtained from a weighted sum of normalized Cu­(Aβ_4–16_) and Cu­(GGH) basis spectra, and the residual shows the difference between the experimental (green line) and reconstructed (dashed line) spectra. The relative contributions of the Cu­(Aβ_4–x_) and Cu­(GGH) basis spectra are used to determine the equilibrium constant $R=K_{\mathrm{Cu}(A\beta_{\mathrm{4‐x}})}^{\prime }/K_{\mathrm{Cu}(\mathrm{GGH})}^{\prime }$ , where the *K*′ values are conditional Cu^2+^ binding constants at pH 7.4, as described in Section 2.5. The vertical scales are different.

Simulations of the room-temperature EPR spectra of moAβ_4–x_ and huAβ_4–x_ in the presence of substoichiometric Cu^2+^ at pH 7.4 (Figure 2A) showed that all are characterized by comparable electron Zeeman (**g**) and copper hyperfine (**A**) parameters (Table 2), indicating that the primary {H_2_N, 2N^–^, N_Im_} coordination environment (Figure 2B) is conserved among these peptides. Room-temperature EPR spectra of Cu^2+^ complexes are characterized not only by their electronic structure but also by their motional dynamics. Therefore, commensurate with their relative molecular weights, Cu^2+^ complexes of Aβ_4–16_ have larger τ_c_ values than those of Aβ_4–9_ (Table 2). Moreover, owing to sequence differences (Table 1), Cu^2+^ complexes of huAβ_4–x_ have slower rotational dynamics (larger τ_c_ values) than those of the corresponding moAβ_4–x_ peptides (Table 2).

**Table 2:** Spectroscopic Parameters for {H_2_N, 2N^–^, N_Im_} (ATCUN) Cu^2+^ Coordination Modes of Murine (mo) and Human (hu) Aβ_4–x_ Peptides and Their Comparison with the Canonical ATCUN Peptide GGH at pH 7.4 (*I* = 0.1 M)^a^

Peptide	UV–vis		EPR				
	λ_max_ (nm)^b^	*ε* (M^–1^ cm^–1^)	*g*_⊥_	*g*_||_	*A*_⊥_ ^c^	*A*_||_ ^c^	τ_c_ ^d^
moAβ_4–9_	515	100	2.036	2.184	24.0	212	0.153
moAβ_4–16_	515	100	2.035	2.179	24.2	210	0.282
huAβ_4–9_	525	100	2.036	2.183	20.9	218	0.195
huAβ_4–16_	525	100	2.035	2.181	20.9	219	0.363
GGH	525	100	2.044	2.178	22.4	216	0.065

a A more detailed comparison with previously obtained parameters and other relevant complexes is shown in Table S1.

b *d–d* band.

c Anisotropic ^65^Cu hyperfine parameters are given in units of 10^–4^ cm^–1^.

d Isotropic rotational correlation time (ns).

Potentiometric studies indicate that multiple {H_2_N, 2N^–^, N_Im_} coordination modes contribute to Cu^2+^ binding above pH 5,^8,9,30^ each with a common first coordination sphere shown in Figure 2B but different outer-sphere ligand protonation states. Thus, each spectrum in Figure 2A strictly quantifies the summation of multiple 1:1 Cu^2+^-bound species at pH 7.4 (cf. eqs 4 and 5), with the single set of **g**, **A**, and τ_c_ parameters in Table 2 being representative of all species within this conditional pool.

Overall, the EPR and UV–vis spectra of the moAβ_4–x_ complexes are similar to those of huAβ_4–x_, indicating that all retain comparable Cu^2+^ coordination properties, with species- and length-dependent differences in axial/second-sphere interactions resulting in only minor differences in the spectroscopic parameters.

### 3.2 Conditional Binding Constants

The ability to delineate spectra of complexes with similar **g** and **A** but different rotational correlation times enabled the Cu^2+^ binding affinity of Aβ_4–x_ peptides to be determined by quantifying their competitive Cu^2+^ binding in the presence of GGH. This canonical ATCUN peptide has well-characterized Cu^2+^ binding affinity^30^ and spectroscopic properties (Table 2), with its low molecular weight (short τ_c_) enabling its EPR spectrum to be distinguished from those of Aβ_4–x_ (Figure 2).

After establishing the time τ required for equilibration of the Cu^2+^ distribution (Section S2, Supporting Information), samples of Cu/Aβ_4–x_/GGH 0.83:1:*n* (*n* = 1–30) were prepared and incubated for a time exceeding 5τ (Figure S5). The EPR spectra were then acquired, and the relative proportions of Cu^2+^ bound to Aβ_4–x_ and GGH (eq 2) were deduced and plotted for each value of *n* = [GGH]_T_/[Aβ_4–x_] (Figure 3).

**Figure 3:**
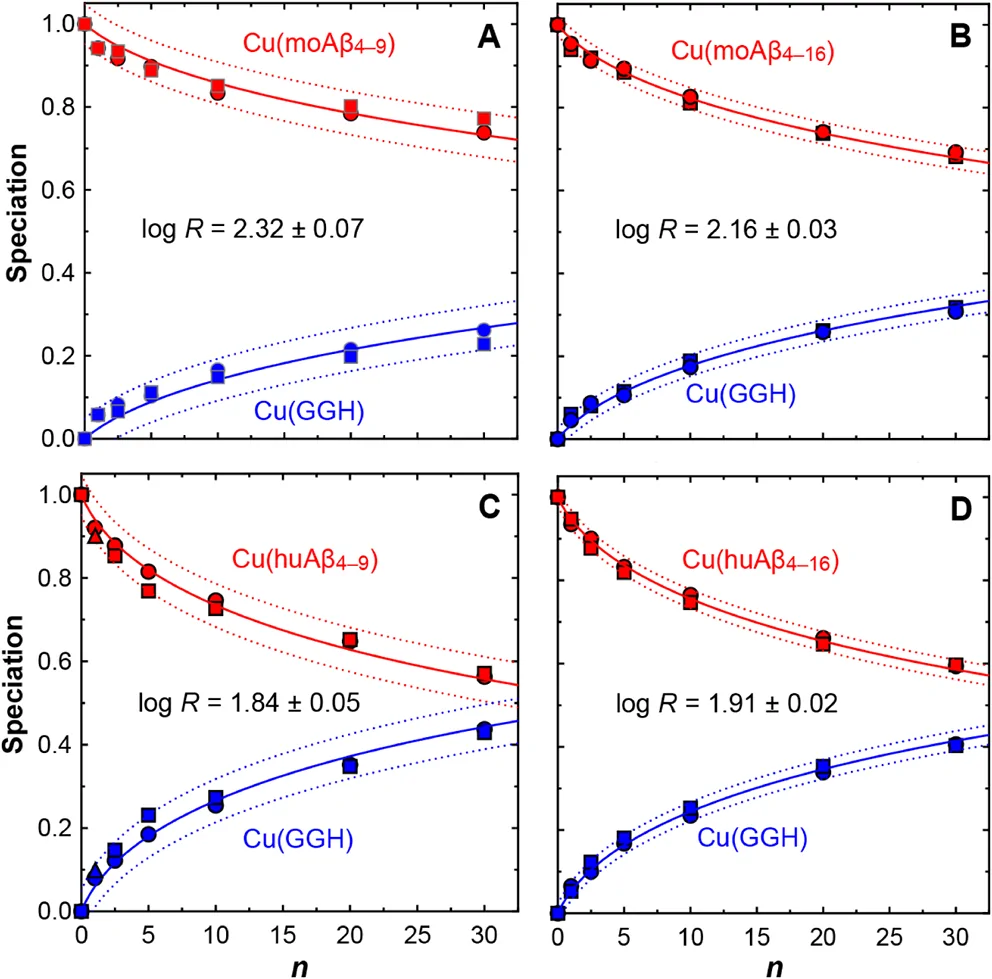
Determination of the relative Cu^2+^ binding affinities of (A) moAβ_4–9_, (B) moAβ_4–16_, (C) huAβ_4–9_, and (D) huAβ_4–16_ in 100 mM NaCl, pH 7.4. Speciation was determined by decomposition (symbols) of the room-temperature EPR spectra of Cu/Aβ_4–x_/GGH 0.83:1:*n* (0 ≤ *n* ≤ 30). Circles and squares show experimental data for two independent sample preparations and analyses. Representative decompositions are shown in Figures S7–S10 for one experimental repeat. Solid lines were calculated using the fitted values of log *R*, where $R=K_{\mathrm{Cu}(A\beta_{\mathrm{4‐x}})}^{\prime }/K_{\mathrm{Cu}(\mathrm{GGH})}^{\prime }$ , and dashed lines enclose the region within which data points are expected.

Nonlinear fitting of the speciation in Figure 3 using eq 8 showed that Aβ_4–x_ peptides have Cu^2+^ binding affinities 1.8–2.3 log units higher than that of GGH at pH 7.4 and thus have log *K*′ values ranging from 14.1 to 14.5 (Table 3). For a given species, the constants for Aβ_4–9_ and Aβ_4–16_ are comparable, whereas the log *K*′ of moAβ_4–x_ is 0.3–0.4 log units higher than that of huAβ_4–x_. With log *K*′ values of 14.4–14.5 (i.e., conditional dissociation constants of 3–4 fM), murine Aβ_4–x_ peptides are the highest-affinity Cu^2+^-binding Aβ species reported to date. Since the competition method relies on differences in rotational correlation times to distinguish the Cu spectra of competing GGH and Aβ_4–x_ peptides, the relative sizes of the uncertainties in log *K*′ (Table 3) are consistent with the relative difference in τ_c_ values between GGH and each Aβ_4–x_ peptide (Table 1), with the largest (smallest) uncertainties being obtained for peptides with τ_c_ values closest to (furthest from) that of GGH.

**Table 3:** Conditional Cu^2+^ Binding Constants (*K*′) of Human and MurineAβ_4–x_ Peptides and the Reference GGH Peptide

Peptide	log [*K*′/(1 M^–1^)]	Reference
moAβ_4–9_	14.5 ± 0.1	This work^a^,^b^
moAβ_4–16_	14.4 ± 0.1	This work^a^,^b^
huAβ_4–9_	14.1 ± 0.1	This work^a^,^b^
	14.183^c^ ± 0.006^d^	^9^
huAβ_4–16_	14.1 ± 0.1	This work^a^,^b^
	13.525^c^ ± 0.007^d^	^8^

a *I* = 0.1 M (NaCl), pH 7.4.

b The formal confidence intervals from nonlinear fitting (Figure 3) account for the scatter in the decomposed speciation data but not possible systematic errors in calculated peptide extinction coefficients or spectral decomposition. Uncertainties were therefore rounded conservatively to ± 0.1 log units.

c *I* = 0.1 M (KNO_3_); conditional constant at pH 7.4 was obtained from potentiometric data using Schwarzenbach coefficients.^33^

d Reflects only formal uncertainties propagated from the tabulated potentiometric constants, assuming independence and ignoring covariance.

Although conditional Cu^2+^ binding constants are pH-dependent, much of this dependence cancels in the GGH competition assay because $\log\;R=\log\;K_{{\mathrm{Cu}(A\beta }_{\mathrm{4‐x}})}^{\prime }-\log\;K_{\mathrm{Cu}(\mathrm{GGH})}^{\prime }$ and both ligands have similar pH-dependent Cu^2+^ binding. For example, using the published potentiometric constants for huAβ_4–x_ ^8,9^ and GGH,^30^ the pH uncertainty Δ­(pH) = 0.03 in the present competition assay would contribute only approximately 0.02 log units to Δ­(log *R*). The estimated contribution of Δ­(pH) to Δ $\left(\log K_{{\mathrm{Cu}(A\beta }_{\mathrm{4‐x}})}^{\prime }\right)$ is therefore smaller than the formal uncertainties obtained from the nonlinear fits.

Although the formal uncertainties in log *R* obtained from the nonlinear fits were smaller than 0.1 log units, the derived absolute conditional constants were rounded conservatively to ± 0.1 log units (Table 3). The fitted uncertainties reflect the scatter of the EPR-derived speciation data and therefore account for random experimental variation, including sample-to-sample pH variation within the accepted range. However, possible systematic errors associated with the calculated peptide extinction coefficients or EPR spectral decomposition may not be reflected in the fitted uncertainties. The ±0.1 log-unit uncertainty should therefore be regarded as a conservative reporting estimate rather than as the formal confidence interval of the nonlinear fit alone.

While the Cu^2+^ binding constants for moAβ_4–x_ peptides have not been previously reported, those for huAβ_4–x_ peptides have been determined using potentiometry.^8,9^ Using the Schwarzenbach coefficients,^33^ the absolute (pH-independent) constants obtained from potentiometry can be converted to conditional (pH-dependent) constants (Table 3). The conditional constant obtained for huAβ_4–9_ in the present study is comparable with that calculated previously.^9^ In contrast, the conditional Cu^2+^ affinity obtained herein for huAβ_4–16_ is 0.6 log units higher than that obtained from stability constants.^8^ Although the absolute conditional constants determined in this study depend on the reference value adopted for Cu­(GGH) and the accuracy of EPR spectral decomposition, the agreement between the present and published values for huAβ_4–9_, together with the comparable affinities obtained here for Aβ_4–9_ and Aβ_4–16_ in both species, suggests that the higher value obtained herein for huAβ_4–16_ is unlikely to arise solely from a systematic error in the competition experiment. The discrepancy may instead reflect the greater model dependence of potentiometric refinements for longer, multi-ionizable peptides such as Aβ_4–16_, in which overlapping protonation equilibria and correlated parameters can make a unique global speciation model more difficult to obtain.

The small but reproducible differences in conditional Cu^2+^ affinity between murine and human Aβ_4–x_ peptides reflect subtle differences in a conserved ATCUN-type CuN_4_ site. Because R5G is the only sequence difference between moAβ_4–9_ and huAβ_4–9_, it must account for the sequence-dependent affinity difference between the Aβ_4–9_ peptides. Since the same substitution is also present in moAβ_4–16_, R5G is the most plausible contributor to the slightly higher conditional Cu^2+^ affinity of the murine peptides. The additional Y10F and H13R substitutions in moAβ_4–16_ may also contribute, but the comparable affinities of moAβ_4–9_ and moAβ_4–16_ suggest the effects of these substitutions are relatively small. Thermodynamically, the difference in Cu^2+^ binding affinity between the mouse and human Aβ_4–x_ peptides reflects a change in the free energy of the Cu-bound ATCUN state relative to the competing apo/protonated peptide ensemble. At the molecular level, replacement of Arg5 by Gly could alter this free-energy difference through changes in outer-sphere hydrogen-bonding, salt bridging, and/or solvation around the CuN_4_ core. However, the magnitude of the effect is small, with a 0.3–0.4 log-unit difference in *K*′ corresponding to |Δ­(Δ*G*′)| = *RT* ln(10) |Δ­(log *K*′)| ≈ 2 kJ mol^–1^ at room temperature. The difference therefore cannot be assigned to a specific microscopic interaction on the basis of the present data but is consistent with modest sequence-dependent changes in the relative free energy of the Cu-bound ATCUN ensemble and the competing apo/protonated peptide ensemble.

### 3.3 ROS Production

The redox activity of Cu­(moAβ_4–x_) complexes in the presence of ascorbate was assessed at pH 7.4 using the APF fluorescence assay of hydroxyl radicals. Neither Cu­(moAβ_4–9_) nor Cu­(moAβ_4–16_) generated significant Cu-mediated ROS compared with the corresponding Cu-free peptide controls. Consistent with previous studies,^8,10^ the corresponding Cu­(huAβ_4–x_) complexes also yielded no significant ROS, whereas peptide-free Cu^2+^ and a Cu­(huAβ_1–16_) positive control both resulted in strong APF responses under the same conditions (Figure 4). The Cu^2+^ complex of the canonical ATCUN peptide GGH also yielded no significant APF signal. Thus, the ATCUN-type complexes do not generate detectable Cu-mediated ROS under the conditions of this assay, consistent with the formation of redox-inactive Cu^2+^ complexes.

**Figure 4:**
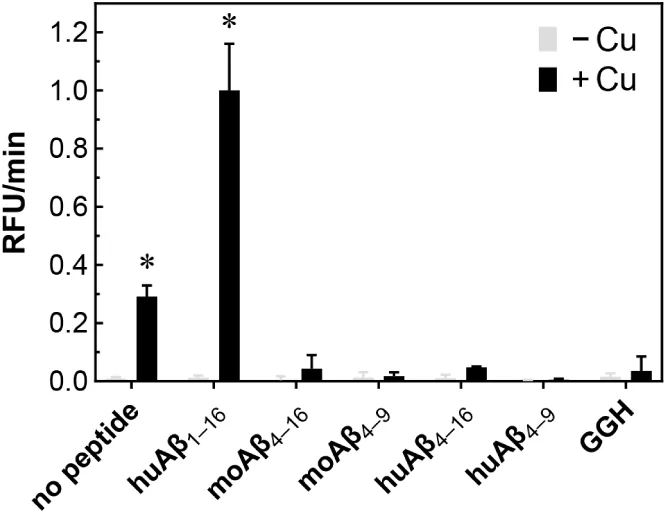
Aminophenyl fluorescein (APF) detection of hydroxyl radical production, showing that ATCUN complexes are redox-silent. The rate of APF fluorescence is normalized relative to the “huAβ_1–16_ + Cu” condition. Initial conditions: [CuCl_2_] = 8 μM, [peptide] = 10 μM, [AscH^–^] = 300 μM, [APF] = 100 μM, [O_2_(aq)] = 270 μM,^35^ [HEPES] = 20 mM, pH 7.4, 25 °C. Mean ± standard deviation is shown. *p < 0.0001 vs all other conditions (two-way ANOVA). Tukey multiple comparisons test results are provided in Table S2. Raw reaction progress curves are shown in Figure S6.

Although HEPES can reportedly scavenge hydroxyl radicals,^34^ its inclusion as a buffer in this assay did not interfere with APF detection of ROS, with the strong responses of peptide-free Cu^2+^ and Cu­(huAβ_1–16_) obtained here in 20 mM HEPES being consistent with those previously obtained in 10 mM phosphate-buffered saline.^8,10^ The results support the conclusion that both murine and human Cu­(Aβ_4–x_) complexes are redox-silent at neutral pH.

## 4 Conclusions

This study showed that murine Aβ_4–x_ peptides retain the defining Cu^2+^ binding properties previously described for the corresponding human Aβ_4–x_ peptides. Both moAβ_4–9_ and moAβ_4–16_ form ATCUN-type Cu^2+^ complexes with a {H_2_N, 2N^–^, N_Im_} first coordination sphere at mildly acidic pH and above. At pH 7.4, the visible absorption and room-temperature EPR spectra of the murine and human complexes show closely comparable spectroscopic parameters, indicating conservation of the primary Cu^2+^ coordination environment despite the R5G, Y10F and H13R sequence differences.

EPR-detected competition with GGH showed that the murine peptides bind Cu^2+^ with low-femtomolar conditional dissociation constants at pH 7.4. The derived values for moAβ_4–9_ and moAβ_4–16_ (3–4 fM) are modestly lower than those measured for the human analogues (8–10 fM), but the Cu^2+^ binding of all four peptides falls within the same high-affinity range. Thus, the R5G substitution within the N-terminal ATCUN site, together with the more distal Y10F and H13R substitutions in moAβ_4–16_, produces only modest second-sphere or conformational effects on the electronic structure and stability of the conserved CuN_4_ core.

The ROS assay further showed that Cu^2+^ complexes of moAβ_4–x_, like those of huAβ_4–x_ and the canonical ATCUN peptide GGH, do not generate Cu-mediated hydroxyl radicals under conditions where those of peptide-free Cu^2+^ and Cu­(huAβ_1–16_) are strongly active. The Cu^2+^ binding of moAβ_4–x_ is therefore both high-affinity and redox-silent.

Together, these results establish that murine Aβ_4–x_ peptides are chemically competent to act as high-affinity, redox-silent Cu^2+^-binding peptides. However, a conserved physiological role for Aβ_4–x_ in Cu^2+^ handling, as previously proposed,^8^ would require not only conservation of the ATCUN-like Cu^2+^ coordination shown here but also generation of Aβ_4–x_ peptides from endogenous APP or Aβ substrates across mammalian species. In humans, while neprilysin-mediated catabolism of huAβ_1–x_ to form huAβ_4–9_ in vivo remains speculative,^24^ huAβ_4–40/42_ is an established major proteoform in the cortex of individuals without diagnosed dementia.^16^ In contrast, endogenous moAβ_4–x_ generation in wild-type mice has not, to our knowledge, been demonstrated. This makes a broadly conserved physiological Cu-binding role possible but currently unsupported.

An alternative interpretation is that Aβ_4–x_ Cu^2+^ binding becomes relevant mainly under pathological conditions. If human amyloid deposits contain peptide-bound Cu^2+^, then the very high Cu^2+^ affinity of the abundant huAβ_4–x_ proteoforms would provide a logical interpretation for such enrichment,^11^ given that they have ∼10^4^-fold higher conditional affinity than huAβ_1–x_ (log *K*′ ∼ 10 at pH 7.4).^36^ The present study does not address possible species-dependent differences in the aggregation behavior of longer Aβ_4–40/42_ peptides, a separate issue from the intrinsic N-terminal Cu^2+^ binding chemistry considered here. However, evidence for Cu “sinking” by Aβ remains equivocal,^11^ with the concept being driven by a longstanding citation bias in the reporting of brain transition metal levels in AD.^37^

A third possibility is that Aβ_4–x_ has no meaningful Cu^2+^ binding role in vivo. Even a peptide with femtomolar Cu^2+^ affinity may be biologically irrelevant if it is not produced at sufficient concentration, is not localized with an exchangeable Cu pool, is rapidly degraded or cleared, or is outcompeted kinetically or thermodynamically by other Cu-binding biomolecules. Direct Cu^2+^ binding to Aβ_4–x_ or indeed any Aβ proteoform in vivo remains unproven, and reports of Cu-bound huAβ_4–40/42_ in biological samples^19^ should also be interpreted cautiously when sample preparation involves low-pH steps that will dissociate native Cu^2+^ complexes and when adventitious Cu^2+^ binding is not controlled.

In summary, the present results show that the moAβ_4–x_ sequence preserves the high-affinity, redox-silent Cu^2+^-binding chemistry of huAβ_4–x_. Thus, moAβ_4–x_ has the bioinorganic capacity required for a Cu^2+^-binding role if it is generated in vivo. However, to our knowledge, there is no evidence that moAβ_4–x_ species are generated from endogenous murine APP/Aβ substrates in wild-type mice. Thus, despite the conserved chemistry, a broadly conserved physiological role for Aβ_4–x_ in mammalian Cu^2+^ handling remains unsupported.

## Supplementary Material



## Notes

Additional experimental details, spectroscopic parameters, supplementary discussion, copper transfer kinetics, statistical analyses, and spectral decompositions (PDF)
