# Supplementary material for: Murine Aβ4–x Peptides Retain High-Affinity Redox-Silent Cu2+ Binding
Source: Inorg Chem. 2026 Oct 23;65(44):26091–100. doi: 10.1021/acs.inorgchem.6c04247 (PMC13753722; doi:10.1021/acs.inorgchem.6c04247)
Supplement: Supplementary file 1 [file ic6c04247_si_001.pdf]

Supporting Information for:

Murine Aβ_{4-x} peptides retain high-affinity redox-silent Cu^{2+} binding

Simon C. Drew

Division of Biophysics, Institute of Experimental Physics, Faculty of Physics

University of Warsaw, 02-093 Warsaw, Poland

E-mail: s.drew@uw.edu.pl

Supplementary Methods

Decomposition of UV-vis and EPR spectra from pH-metric titrations of Cu/Aβ$_{4-x}$

UV-vis spectra were analysed as-is, while EPR spectra were normalized by double integration prior to analysis. Basis spectra corresponding to {6H_2O} (aqueous Cu^{2+}) and {H_2N, 2N^-, N_{Im}} (ATCUN) were obtained from spectra at pH < 3 and pH > 7, respectively. This basis set was then used to reconstruct the spectra across the full range of pH values, the weightings being iteratively varied using a Generalized Reduced Gradient Nonlinear solver (Frontline Systems, Inc., www.solver.com) to minimize the mean-squared deviation between the reconstructions and the experimental spectra. The intensity of each experimental EPR spectrum was permitted to be varied by ±5% to allow for normalisation errors, and the vertical position of each experimental UV-vis spectrum was permitted to be varied by ±0.003 to allow for baseline offset errors. GraphPad Prism was used to fit an inbuilt five-parameter asymmetric sigmoidal function to the experimental speciation of {6H_2O} and {H_2N, 2N^-, N_{Im}} and to fit a user-defined split-width gaussian function ($\sigma = \sigma_{left}$ when $X < X_0$; $\sigma = \sigma_{right}$ when $X > X_0$) to the experimental speciation of {H_2N, N_{Im}, 2O} (*vide infra*).

Supplementary Discussion

S1. Decomposition of UV-vis and EPR spectra from pH-metric titrations of Cu/Aβ$_{4-x}$

Consistent with the absence of a clear isosbestic point between the absorption lines of {6H_2O} (λ_{max} ≈ 815 nm) and {H_2N, 2N^-, N_{Im}} (λ_{max} ≈ 515 nm) species, decomposition of spectra in the pH 3–5 range resulted in large deviations between the reconstructions and the experimental spectra when only basis spectra corresponding to {6H_2O} and {H_2N, 2N^-, N_{Im}} were used. Indeed, an additional species with a peak concentration at pH ≈ 4 could be isolated (Figures S1–S2), and the isolated absorption line corresponding to this species (λ_{max} = 725 nm, ε = 40 $M^{-1}cm^{-1}$) was ascribed to a {H_2N, N_{Im}, 2O}

coordination mode (Table S1). Although the algebraic isolation of the {H_2N, N_{Im}, 2O} species (Figures S1–S2) requires a judicious choice of weightings, the inclusion of this species was required to properly decompose the electronic absorption and EPR spectra in the low-pH range (Figure 1, main text). Simulations of the room-temperature EPR spectra of the isolated species were not attempted owing to the low signal-to-noise ratio. Although **g** and **A** parameters can more easily be obtained from frozen-solution spectra, this minor species does not affect the Cu binding properties of these peptides above pH 5.

The presence of a 2N2O species for Cu/moAβ_{4-x} systems is not unexpected. In our previous potentiometric analysis of the canonical ATCUN peptide, GGH (on which the conditional Cu^{2+} binding constants of Aβ_{4-x} peptides in this study rely), there was no clear isosbestic point between the absorption lines of {$6H_2O$} ($\lambda_{max} \approx 815$ nm) and {H_2N, $2N^-$, N_{Im}} ($\lambda_{max} \approx 525$ nm) species [1]. This is consistent with the presence of an additional species, which was assigned to a {H_2N, N_{Im}, 2O} coordination mode, with a peak absorption at an intermediate wavelength. Potentiometry indicated that this coordination mode has a peak speciation in the pH 4–5 range, and this was corroborated by the pH dependence of the change in absorbance at 685 nm across the pH 3.5–5.5 range [1]. To demonstrate this more clearly, the UV-vis spectra from ref. [1] were reanalysed. Figure S3 shows the isolation of the absorption line corresponding to {H_2N, N_{Im}, 2O} ($\lambda_{max} = 705$ nm, $\varepsilon = 40$ $M^{-1}cm^{-1}$), together with the plot of Cu speciation obtained by decomposition of the pH-metric UV-vis spectra. The λ_{max} and ε values of the spectrum assigned to {H_2N, N_{Im}, 2O} (Table S1) are compatible with a 2N2O coordination environment.

The fact that the canonical ATCUN peptide GGH forms a {H_2N, N_{Im}, 2O} species at intermediate pH suggests that other ATCUN peptides should do the same. Thus, it is not surprising that we isolated such a species in Cu/moAβ_{4-x} systems and that it has been identified in other Cu/ATCUN systems [2]. For Cu/huAβ_{4-x} systems, previous potentiometric models did not include a {H_2N, N_{Im}, 2O} species despite a related low-pH signal being observed in frozen-solution EPR spectra and there being no sharp isosbestic point between the absorption lines of {$6H_2O$} ($\lambda_{max} \approx 815$ nm) and {H_2N,

$2N^{-}$, N_{Im}} (λ_{max} ≈ 525 nm) species [3]. Instead, the intermediate-pH species observed in the EPR spectra was attributed to a freezing-induced artefact. Considering the present observation of an intermediate-pH species in Cu/moAβ_{4-x} systems (Figure 2, main text) and its presence in other Cu/ATCUN systems including [1,2], this feature can be concluded to be a genuine minor species that was not included in the potentiometric models of huAβ_{4-x}. Analysis of the frozen-solution EPR spectra (Figure S4A) from the previous pH-metric study of Cu/huAβ_{4-9} [3] enabled isolation of the intermediate-pH signal (Figure S4B,C) and determination of a pH-dependent speciation (Figure S4D) that is qualitatively consistent with that observed for Cu/moAβ_{4-x} systems (Figure 2, main text), although the peak speciation of ≈35% at 77 K greatly exceeds that observed for the Cu/GGH and Cu/moAβ_{4-x} systems at room temperature. Indeed, scrutiny of the published pH-metric UV-vis spectra of Cu/huAβ_{4-x} suggests that, if present, the intermediate-pH complex – putatively a {H_2N, N_{Im}, 2O} coordination mode, as proposed for other Cu/ATCUN systems – has a peak speciation of <10%. Thus, frozen-solution EPR likely shifts the ratio of {H_2N, N_{Im}, 2O} and {H_2N, $2N^{-}$, N_{Im}} speciation rather than introducing "freezing artefacts". Although the published potentiometric models for Cu/huAβ_{4-x} systems would require refinement to account for the minor intermediate-pH species, it would not alter the conditional Cu^{2+} binding constants at pH 7.4 because at this pH the intermediate-pH species is not present.

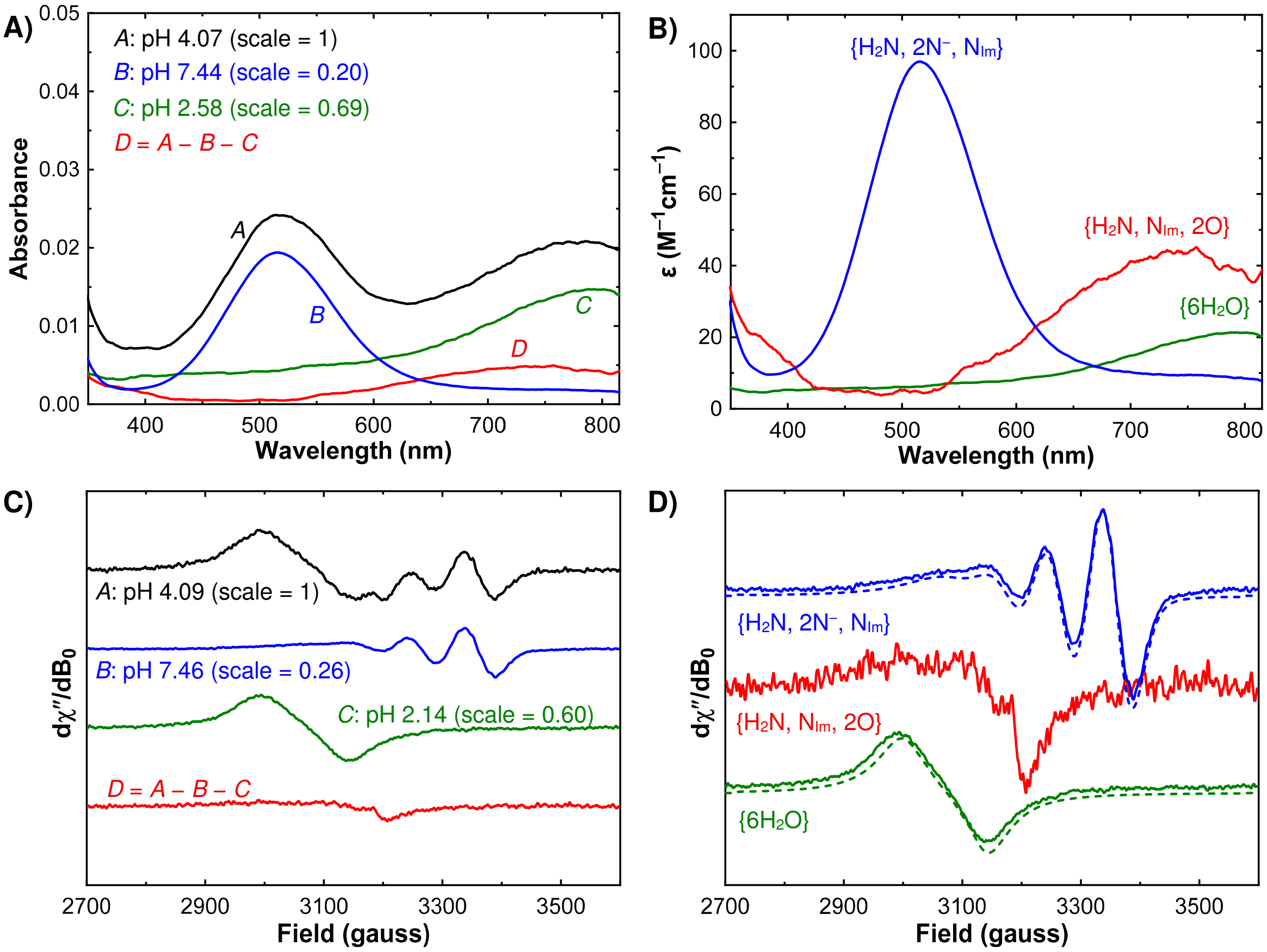

Figure S1. Isolation of basis spectra used for analysis of pH-metric titrations of ^{65}Cu/moA$\beta_{4–9}$ 0.8:1 in 100 mM NaCl, 25°C ($[Cu]_T$ = 1 mM). **(A)** Algebraic isolation of the absorption spectrum of an intermediate-pH mixed N/O species putatively assigned to a {H_2N, N_{Im}, 2O} coordination mode. **(B)** Basis set of spectra corresponding to a {H_2N, $2N^-$, N_{Im}} species (ATCUN), a {$6H_2O$} species (aquated Cu^{2+}), and the putative {H_2N, N_{Im}, 2O} species isolated in panel *A*. **(C)** Algebraic isolation of the room-temperature EPR spectrum of a 2N2O coordination mode at pH ≈ 4. **(D)** Basis set of spectra corresponding to a {H_2N, $2N^-$, N_{Im}} species, {$6H_2O$} species (aquated Cu^{2+}), and the putative {H_2N, N_{Im}, 2O} species isolated in panel *C*. Dashed lines show simulations (Section 2.4.3, main text) obtained using the parameters in Table S1. The scales in panels *C* and *D* are different.

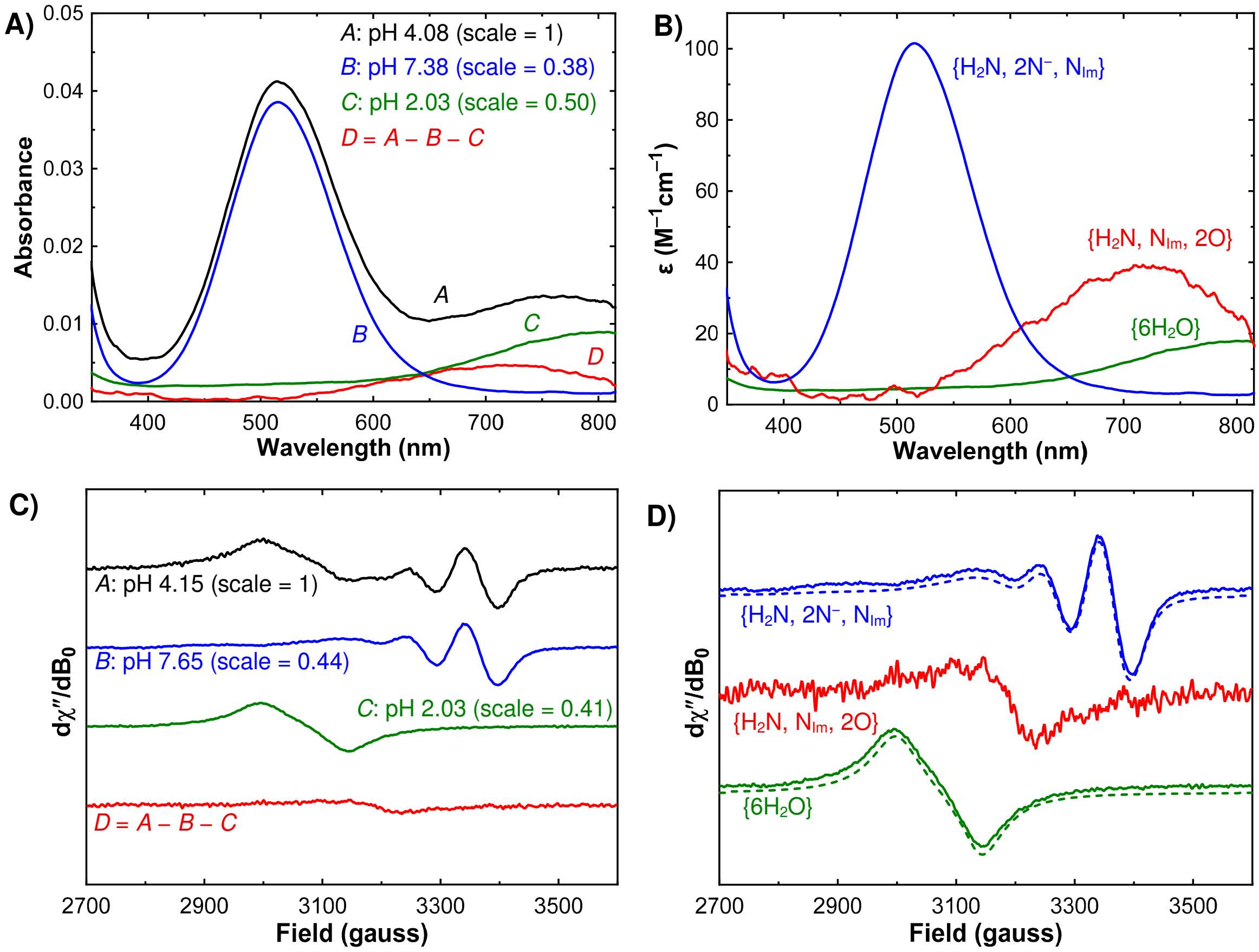

Figure S2. Isolation of basis spectra used for analysis of pH-metric titrations of ^{65}Cu/moAβ$_{4–16}$ 0.8:1 in 100 mM NaCl, 25°C ([Cu]$_T$ = 1 mM). **(A)** Algebraic isolation of the absorption spectrum of an intermediate-pH mixed N/O species putatively assigned to a {H_2N, N_{Im}, 2O} coordination mode. **(B)** Basis set of spectra corresponding to a {H_2N, $2N^-$, N_{Im}} species (ATCUN), a {$6H_2O$} species (aquated Cu^{2+}), and the putative {H_2N, N_{Im}, 2O} species isolated in panel *A*. **(C)** Algebraic isolation of the room-temperature EPR spectrum of a 2N2O coordination mode at pH ≈ 4. **(D)** Basis set of spectra corresponding to a {H_2N, $2N^-$, N_{Im}} species, {$6H_2O$} species (aquated Cu^{2+}), and the putative {H_2N, N_{Im}, 2O} species isolated in panel *C*. Dashed lines show simulations (Section 2.4.3, main text) using the parameters in Table S1. The scales in panels *C* and *D* are different.

Table S1. Spectroscopic parameters for Cu^{2+} complexes of $A\beta_{4\text{–}x}$ and GGH. (n.d. = not determined; n.a. = not applicable).

**Peptide**	**UV–vis** [a]		**EPR** [b]									**Reference**
	λ_{max}	ε	$g_{\perp}$	$g_{\parallel}$	$A_{\perp}$	$A_{\parallel}$	a^{NH_2}	$a^{N_{Im}}$	a^{N^-}	a^{N^-}	τ_c [c]	
**mo$A\beta_{4\text{–}9}$**												
{H_2N, N_{Im}, 2O} [d]	735	40	n.d.	n.d.	n.d.	n.d.	n.d.	n.d.	n.a.	n.a.	n.d.	This work
{H_2N, $2N^-$, N_m}	515	100	2.034	2.187	23.5	212	n.d.	n.d.	n.d.	n.d.	0.141	This work
**mo$A\beta_{4\text{–}16}$**												
{H_2N, N_{Im}, 2O} [d]	720	40	n.d.	n.d.	n.d.	n.d.	n.d.	n.d.	n.a.	n.a.	n.d.	This work
{H_2N, $2N^-$, N_{Im}}	515	100	2.035	2.179	24.2	210	n.d.	n.d.	n.d.	n.d.	0.282	This work
**hu$A\beta_{4\text{–}9}$**												
{H_2N, N_{Im}, 2O} [d]	n.d.	n.d.	2.069	2.343	10.1	168	n.d.	n.d.	n.a.	n.a.	Frozen	This work, [3]
{H_2N, $2N^-$, N_{Im}}	525	100	2.043 [e]	2.184	19.9 [e]	214	n.d.	n.d.	n.d.	n.d.	Frozen	This work, [3]
			2.036	2.183	20.9	218	n.d.	n.d.	n.d.	n.d.	0.195	This work
**hu$A\beta_{4\text{–}16}$**												
{H_2N, $2N^-$, N_{Im}}	525	100	2.043	2.183	25	215	n.d.	n.d.	n.d.	n.d.	Frozen	[4]
			2.035	2.181	20.9	219	n.d.	n.d.	n.d.	n.d.	0.363	This work
**GGH**												
{H_2N, N_{Im}, 2O} [d]	705	40	n.d.	n.d.	n.d.	n.d.	n.d.	n.d.	n.a.	n.a.	n.d.	This work, [1]
	705	48	n.d. [f]	n.d. [f]	n.d. [f]	n.d. [f]	n.d. [f]	n.d. [f]	n.a.	n.a.	Frozen	[5]
{H_2N, $2N^-$, N_{Im}}	525	100	2.044	2.178	22.4	216	10.8	12.1	14.8	14.8	0.065	This work
			2.044	2.177	21.3	217	11.0	11.8	14.6	15.1	0.063	[1]
**H_2O**												
{$6H_2O$}	815	12.6	2.088	2.401	−14	139	n.a.	n.a.	n.a.	n.a.	0.004	This work
			2.081	2.413	−10	145	n.a.	n.a.	n.a.	n.a.	Frozen	This work, [3]

[a] λ_{max} (nm), ε ($M^{-1}cm^{-1}$).

[b] Anisotropic ^{65}Cu hyperfine (A) and isotropic ^{14}N ligand hyperfine (a) parameters are in units of 10^{-4} cm^{-1}.

[c] Isotropic rotational correlation time (ns).

[d] Putative assignment of the intermediate-pH mixed N/O species.

[e] **g** and **A** are rhombic; $g_{\perp} = (g_x + g_y)/2$ with $g_x = 2.033$, $g_y = 2.053$; $A_{\perp} = (A_x + A_y)/2$ with $A_x = 13.8$, $A_y = 26.0$.

[f] Spectra obtained in 200 mM acetate buffer pH 4.5 appear to contain monomeric Cu(acetate)$_2$ [6] artefacts.

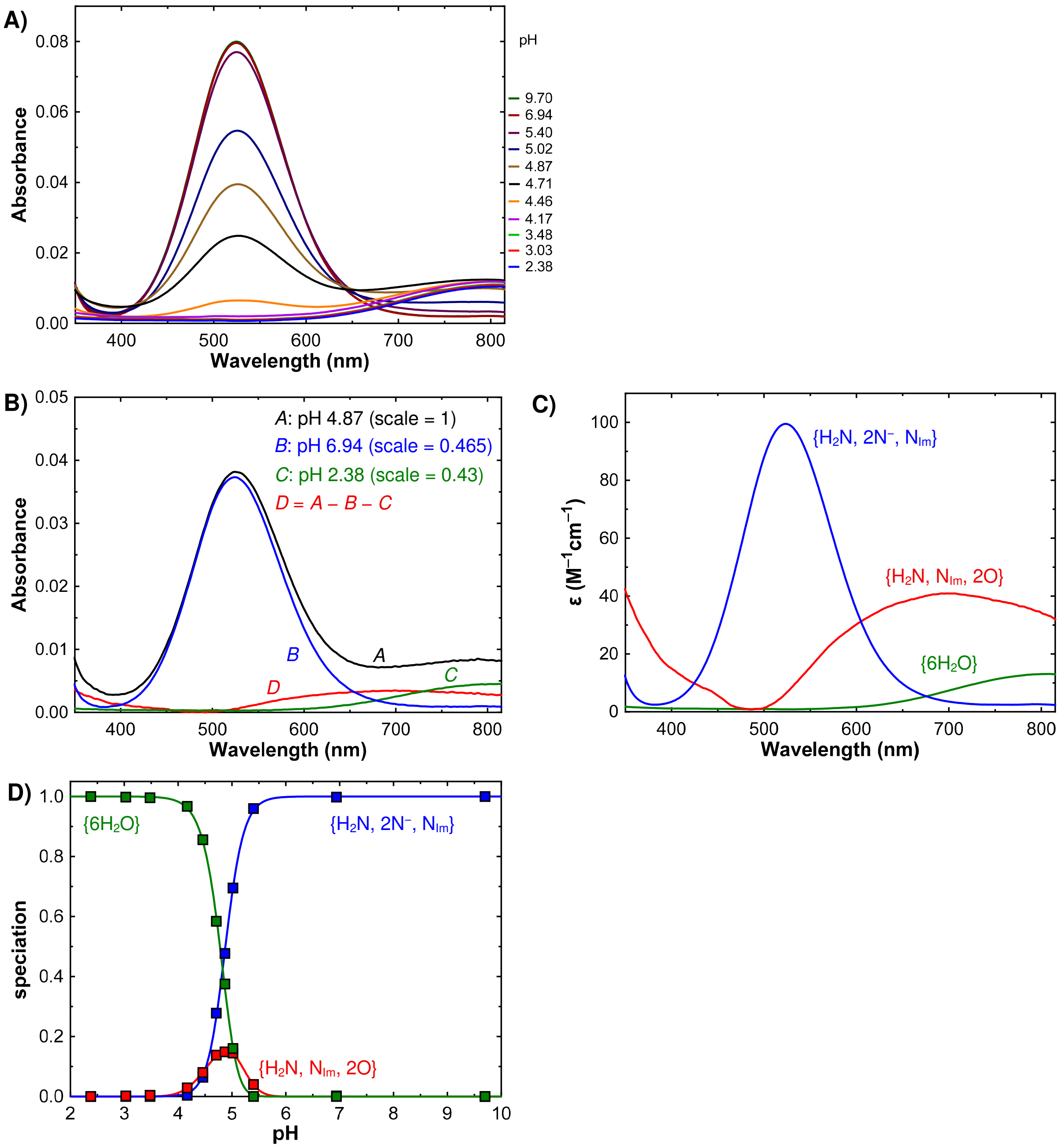

Figure S3. UV-vis spectra of ^{65}Cu/GGH 0.8:0.95 at 25°C (I = 0.1 M, $[Cu]_T$ = 0.8 mM) reproduced from ref. [1]. **(A)** pH-metric titration. **(B)** Algebraic isolation of the absorption spectrum of a intermediate-pH mixed N/O species putatively assigned to a $\{H_2N, N_{Im}, 2O\}$ coordination mode. **(C)** Basis set of spectra corresponding to a $\{H_2N, 2N^-, N_{Im}\}$ species, $\{6H_2O\}$ species (aquated Cu^{2+}), and the putative $\{H_2N, N_{Im}, 2O\}$ species isolated in panel *B*. **(D)** Cu speciation determined by decomposition of the spectra in panel *A* using the basis set in panel *C*.

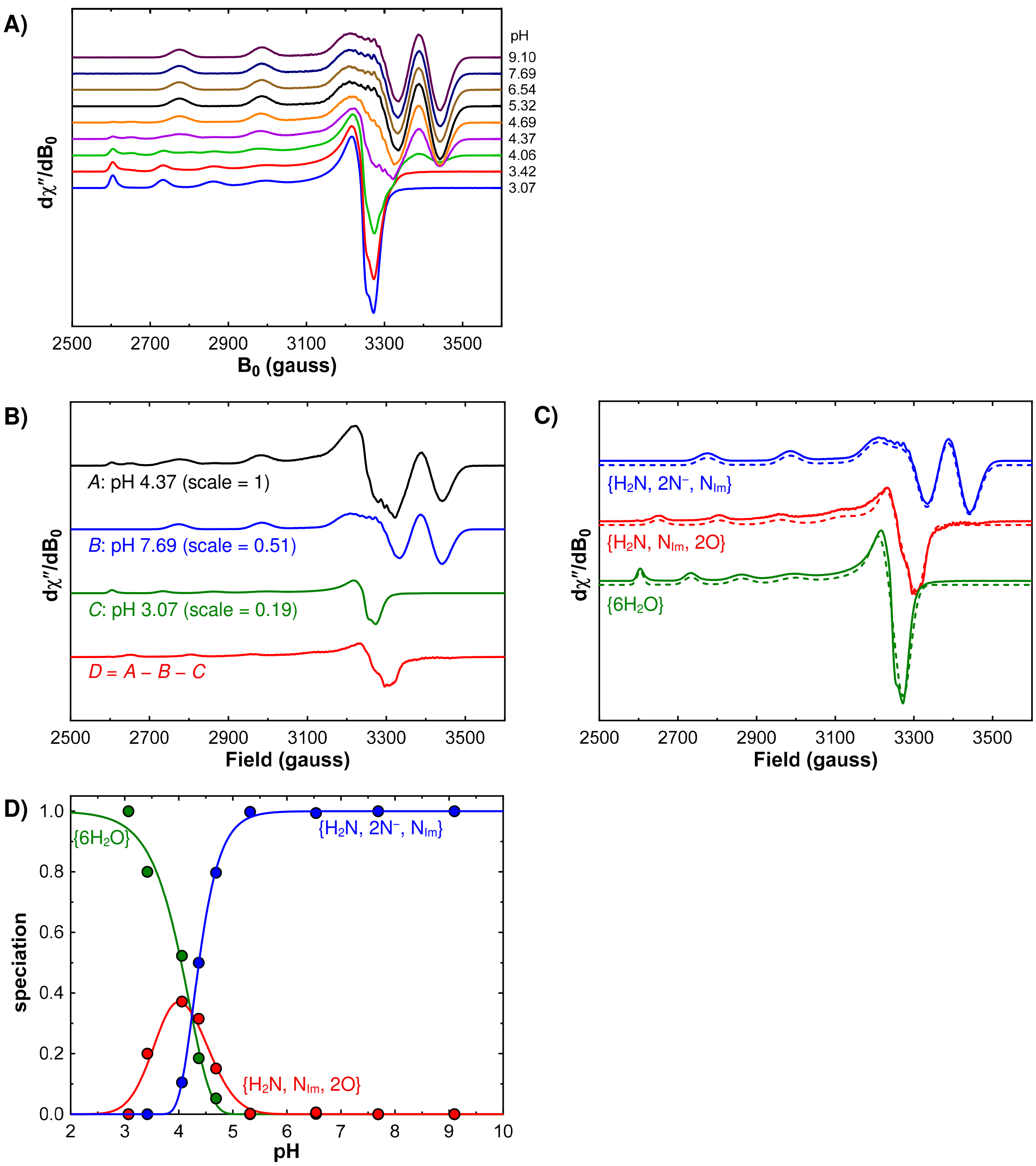

Figure S4. Analysis of frozen-solution (77 K) X-band (9.450 GHz) EPR spectra of ^{65}Cu/huA$\beta_{4\text{–}9}$ 0.8:1 ($[Cu]_T$ = 0.8 mM) reproduced from ref. [3]. **(A)** pH-metric titration. **(B)** Algebraic isolation of the EPR spectrum of an intermediate-pH mixed N/O species putatively assigned to a {H_2N, N_{Im}, 2O} coordination mode. **(C)** Basis set of spectra corresponding to a {H_2N, $2N^-$, N_{Im}} species, {$6H_2O$} species (aquated Cu^{2+}), and the putative {H_2N, N_{Im}, 2O} species isolated in panel *B*. Dashed lines show simulations using the "pepper" function with matrix diagonalisation in EasySpin v.6.0.6 [7] and the parameters in Table S1. **(D)** Cu speciation determined by decomposition of the spectra in panel *A* using the basis set in panel *C*. The scales in panels *A*–*C* are different.

S2. Cu^{2+} transfer kinetics

To measure the relative Cu^{2+} binding affinity of $A\beta_{4-x}$ and GGH peptides, an equilibrium Cu^{2+} species distribution is required. Previous studies of competition between Aβ peptides with different ATCUN motifs indicated that metal-ion exchange in the absence of other Cu^{2+}-binding molecules takes place on the timescale of days [3]. Therefore, to estimate the time required for equilibrium be established, GGH was added to Cu/$A\beta_{4-x}$ 0.83:1 to achieve a final molar ratio of Cu/Aβ/GGH 0.83:1:10, and EPR spectra were then acquired as a function of time post mixing. For each time point, the relative proportions of Cu^{2+} bound to $A\beta_{4-x}$ and GGH (Equation 2) were deduced, as exemplified in Figure 2B (main text). Then the proportions were plotted as a function of time.

As shown in Figure S5, the time constant for Cu transfer from $A\beta_{4-x}$ to GGH ranged from approximately 1–3 days. The murine $A\beta_{4-9}$ and $A\beta_{4-16}$ peptides showed similar Cu-transfer kinetics to excess GGH, whereas the human peptides differed more substantially, with hu$A\beta_{4-16}$ showing faster transfer than hu$A\beta_{4-9}$. The spectroscopic parameters indicate that all $A\beta_{4-x}$ complexes retain broadly similar ATCUN-based Cu^{2+} coordination environments (Table S1). Therefore, the different Cu-transfer kinetics likely reflect subtle differences in local conformation, axial/second-sphere interactions, or accessibility of the Cu^{2+} centre to GGH. Although these data do not by themselves define the coordination geometry, the trend is consistent with the possibility that extension beyond Gly9 perturbs human-specific intramolecular interactions involving Arg5 and the C-terminal region of the short $A\beta_{4-9}$ fragment.

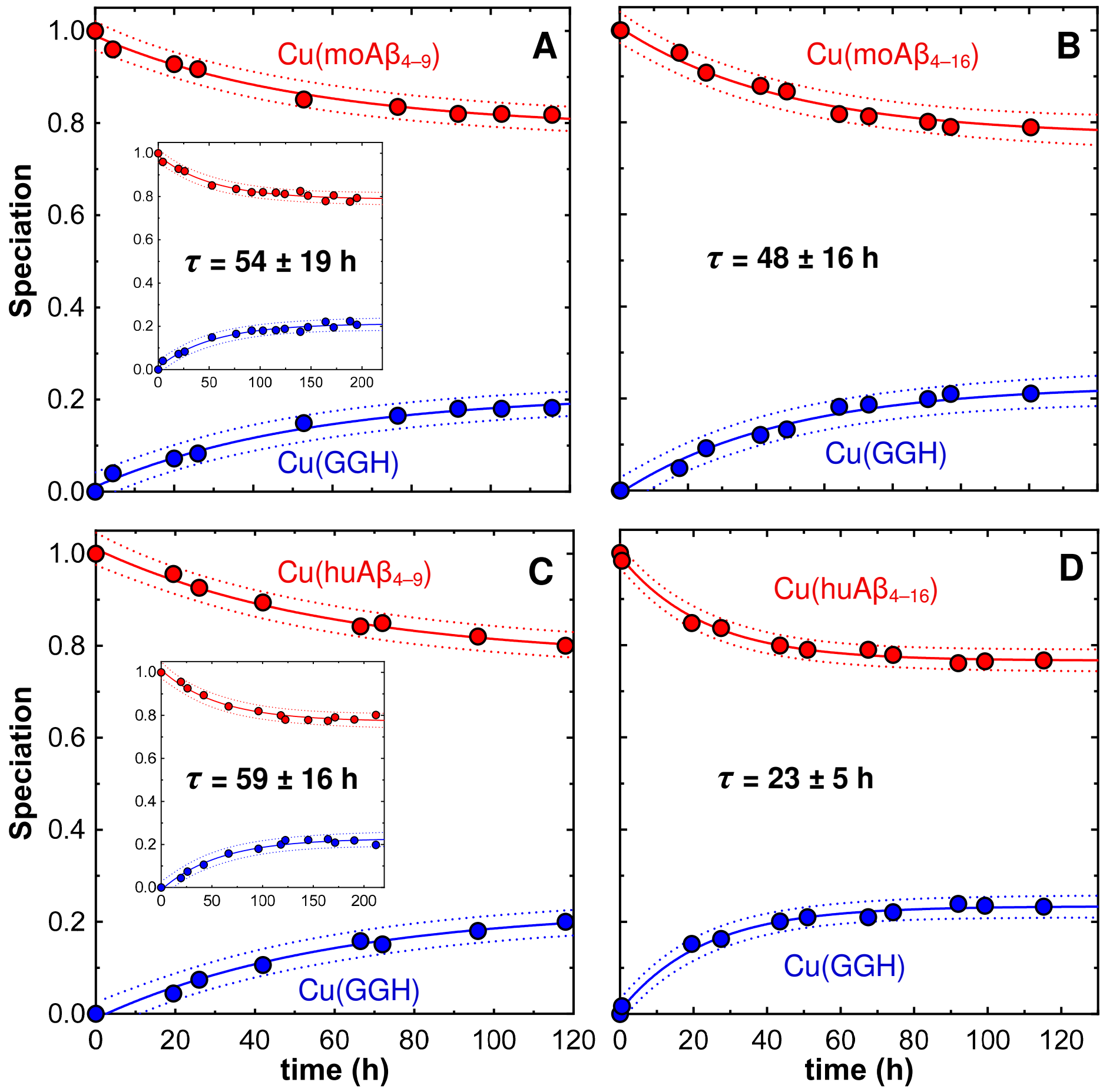

Figure S5. Kinetics of Cu^{2+} transfer from **(A)** moA$\beta_{4–9}$, **(B)** moA$\beta_{4–16}$, **(C)** huA$\beta_{4–9}$, and **(D)** huA$\beta_{4–16}$ to GGH. Samples of ^{65}Cu/A$\beta_{4–x}$ 0.83:1 (1.2 mM Aβ) and 12 mM GGH were prepared in 100 mM NaCl, pH 7.4. For each A$\beta_{4–x}$ peptide, equal volumes of ^{65}Cu/A$\beta_{4–x}$ 0.83:1 (1.2 mM A$\beta_{4–x}$) and 12 mM GGH were then combined to yield a final stoichiometry of Cu/A$\beta_{4–x}$/GGH 0.83:1:10 (0.5 mM ^{65}Cu), and EPR spectra were acquired at various times after mixing. Percentages of Cu(A$\beta_{4–x}$) and Cu(GGH) were determined by decomposing the spectra of Cu/A$\beta_{4–x}$/GGH into separate components using Cu/A$\beta_{4–x}$ 0.83:1 and Cu/GGH 0.83:1 as basis spectra. Dashed lines enclose the region within which 95% of additional data points are expected.

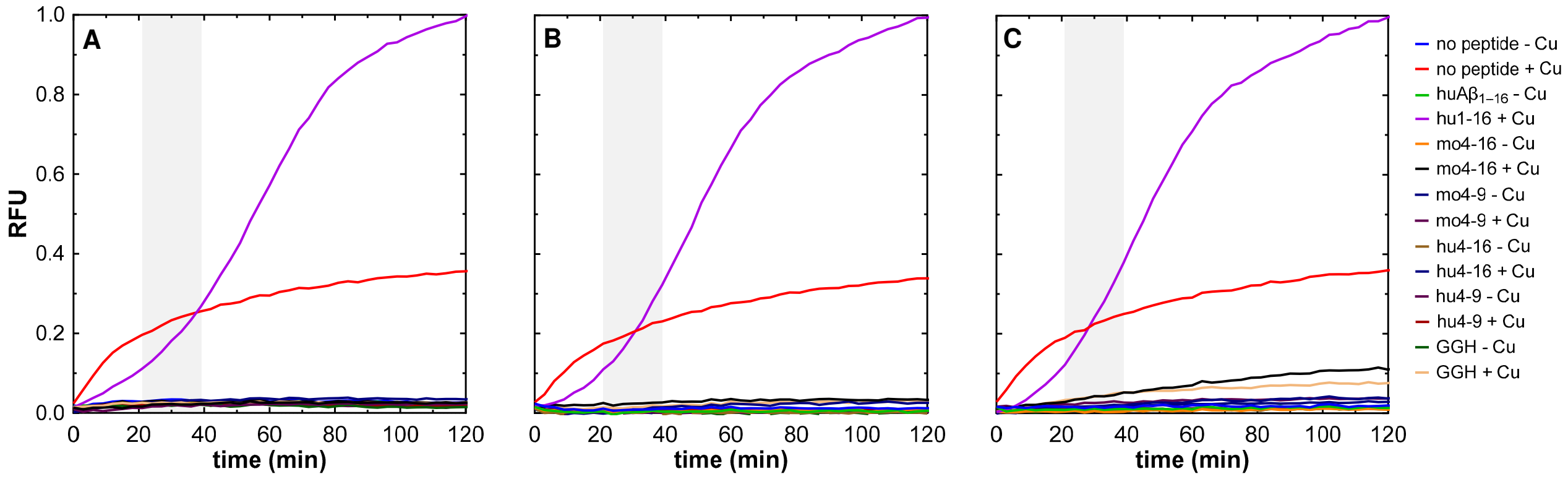

Figure S6. Raw reaction progress curves showing APF fluorescence for three independent experiments prepared and performed on different days. The ROS rates plotted in Figure 4 (main text) were obtained from linear fits in the shaded region. **(A)** $n = 1$. **(B)** $n = 2$. **(C)** $n = 3$.

Table S2. Multiple comparisons tests for the ROS assay data shown in Figure 4 (main text).

Number of families	9
Number of comparisons per row family	1
Number of comparisons per column family	21
Alpha	0.05

Tukey's multiple comparisons test	Mean Diff.	95.00% CI of diff.	Below threshold?	Summary	Adjusted P Value
no peptide					
-Cu vs. +Cu	-0.282	-0.363 to -0.200	Yes	****	<0.0001
huAβ_{1-16}					
-Cu vs. +Cu	-0.988	-1.07 to -0.907	Yes	****	<0.0001
moAβ_{4-16}					
-Cu vs. +Cu	-0.0403	-0.122 to 0.0410	No	ns	0.3189
moAβ_{4-9}					
-Cu vs. +Cu	-0.00386	-0.0851 to 0.0774	No	ns	0.9232
huAβ_{4-16}					
-Cu vs. +Cu	-0.0372	-0.118 to 0.0440	No	ns	0.3562
huAβ_{4-9}					
-Cu vs. +Cu	-0.00729	-0.0885 to 0.0740	No	ns	0.8555
GGH					
-Cu vs. +Cu	-0.0201	-0.101 to 0.0612	No	ns	0.6167
-Cu					
no peptide vs. huAβ_{1-16}	-0.00289	-0.129 to 0.123	No	ns	>0.9999
no peptide vs. moAβ_{4-16}	0.00647	-0.119 to 0.132	No	ns	>0.9999
no peptide vs. moAβ_{4-9}	-0.00369	-0.130 to 0.122	No	ns	>0.9999
no peptide vs. huAβ_{4-16}	-0.00107	-0.127 to 0.125	No	ns	>0.9999
no peptide vs. huAβ_{4-9}	0.0118	-0.114 to 0.138	No	ns	>0.9999
no peptide vs. GGH	-0.00609	-0.132 to 0.120	No	ns	>0.9999
huAβ_{1-16} vs. moAβ_{4-16}	0.00936	-0.116 to 0.135	No	ns	>0.9999
huAβ_{1-16} vs. moAβ_{4-9}	-0.000799	-0.127 to 0.125	No	ns	>0.9999
huAβ_{1-16} vs. huAβ_{4-16}	0.00182	-0.124 to 0.128	No	ns	>0.9999
huAβ_{1-16} vs. huAβ_{4-9}	0.0147	-0.111 to 0.141	No	ns	0.9998
huAβ_{1-16} vs. GGH	-0.00320	-0.129 to 0.123	No	ns	>0.9999
moAβ_{4-16} vs. moAβ_{4-9}	-0.0102	-0.136 to 0.116	No	ns	>0.9999
moAβ_{4-16} vs. huAβ_{4-16}	-0.00754	-0.133 to 0.118	No	ns	>0.9999
moAβ_{4-16} vs. huAβ_{4-9}	0.00534	-0.120 to 0.131	No	ns	>0.9999
moAβ_{4-16} vs. GGH	-0.0126	-0.138 to 0.113	No	ns	>0.9999
moAβ_{4-9} vs. huAβ_{4-16}	0.00262	-0.123 to 0.128	No	ns	>0.9999
moAβ_{4-9} vs. huAβ_{4-9}	0.0155	-0.110 to 0.141	No	ns	0.9997
moAβ_{4-9} vs. GGH	-0.00241	-0.128 to 0.123	No	ns	>0.9999
huAβ_{4-16} vs. huAβ_{4-9}	0.0129	-0.113 to 0.139	No	ns	0.9999
huAβ_{4-16} vs. GGH	-0.00503	-0.131 to 0.121	No	ns	>0.9999
huAβ_{4-9} vs. GGH	-0.0179	-0.144 to 0.108	No	ns	0.9992
+Cu					
no peptide vs. huAβ_{1-16}	-0.709	-0.835 to -0.583	Yes	****	<0.0001
no peptide vs. moAβ_{4-16}	0.248	0.122 to 0.374	Yes	****	<0.0001
no peptide vs. moAβ_{4-9}	0.274	0.148 to 0.400	Yes	****	<0.0001
no peptide vs. huAβ_{4-16}	0.243	0.118 to 0.369	Yes	****	<0.0001
no peptide vs. huAβ_{4-9}	0.286	0.160 to 0.412	Yes	****	<0.0001
no peptide vs. GGH	0.255	0.130 to 0.381	Yes	****	<0.0001
huAβ_{1-16} vs. moAβ_{4-16}	0.957	0.831 to 1.08	Yes	****	<0.0001
huAβ_{1-16} vs. moAβ_{4-9}	0.983	0.857 to 1.11	Yes	****	<0.0001
huAβ_{1-16} vs. huAβ_{4-16}	0.952	0.827 to 1.08	Yes	****	<0.0001
huAβ_{1-16} vs. huAβ_{4-9}	0.995	0.869 to 1.12	Yes	****	<0.0001
huAβ_{1-16} vs. GGH	0.965	0.839 to 1.09	Yes	****	<0.0001
moAβ_{4-16} vs. moAβ_{4-9}	0.0262	-0.0996 to 0.152	No	ns	0.9937
moAβ_{4-16} vs. huAβ_{4-16}	-0.00449	-0.130 to 0.121	No	ns	>0.9999
moAβ_{4-16} vs. huAβ_{4-9}	0.0383	-0.0875 to 0.164	No	ns	0.9573
moAβ_{4-16} vs. GGH	0.00761	-0.118 to 0.133	No	ns	>0.9999
moAβ_{4-9} vs. huAβ_{4-16}	-0.0307	-0.157 to 0.0951	No	ns	0.9856
moAβ_{4-9} vs. huAβ_{4-9}	0.0121	-0.114 to 0.138	No	ns	>0.9999
moAβ_{4-9} vs. GGH	-0.0186	-0.144 to 0.107	No	ns	0.9991
huAβ_{4-16} vs. huAβ_{4-9}	0.0428	-0.0830 to 0.169	No	ns	0.9291
huAβ_{4-16} vs. GGH	0.0121	-0.114 to 0.138	No	ns	>0.9999
huAβ_{4-9} vs. GGH	-0.0307	-0.157 to 0.0951	No	ns	0.9857

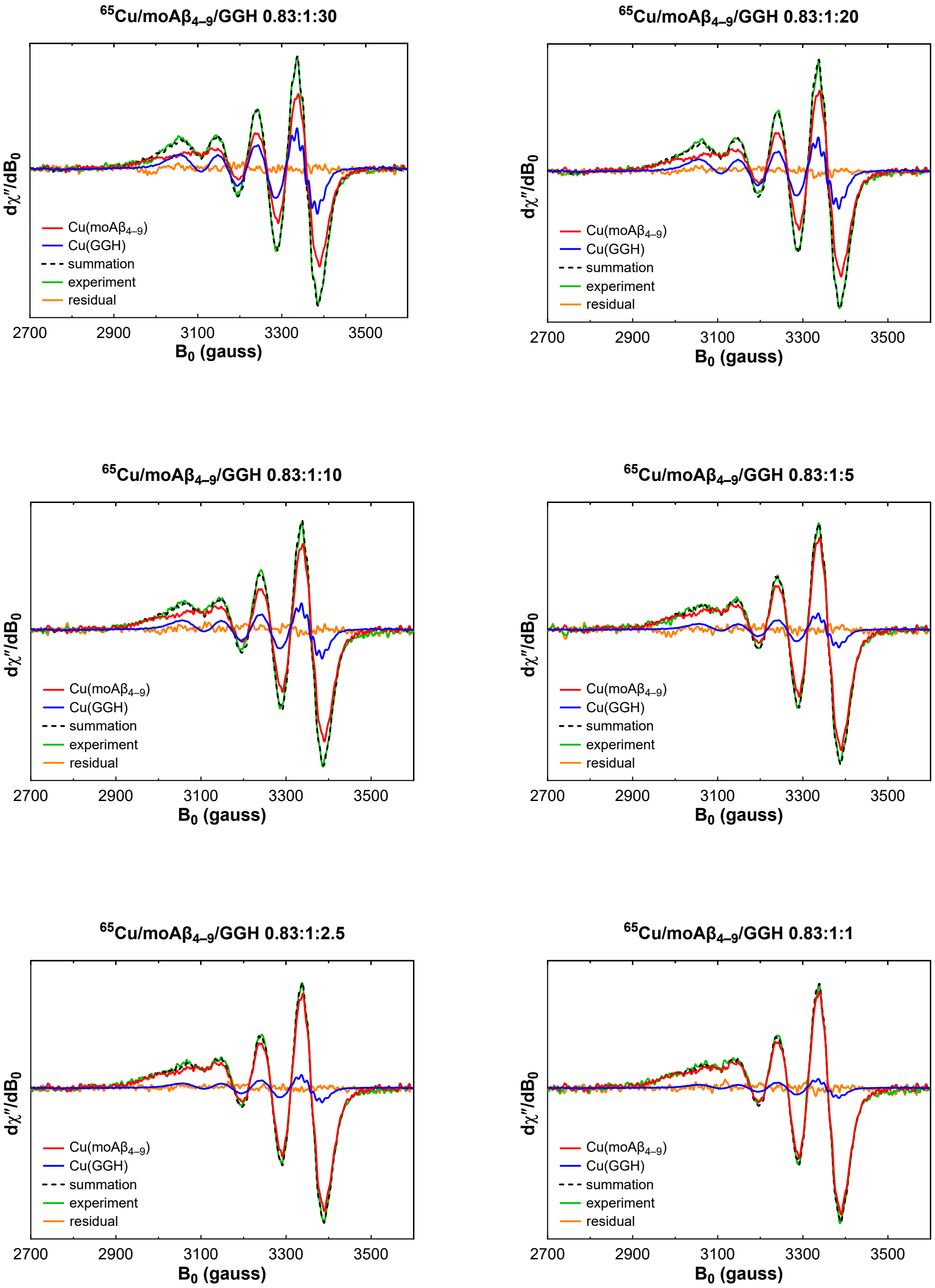

Figure S7. Decomposition of the room-temperature X-band (9.42 GHz) EPR spectra of Cu/moAβ$_{4–9}$/GGH mixtures prepared at molar ratios of $0.83 : 1 : n$, where $n = [\mathrm{GGH}]_T / [\mathrm{moA\beta_{4–9}}]_T =$ 1–30. Percentages of each species are plotted in Figure 3A (main text) and were determined by minimising the difference (*orange*) between the experimental spectrum (*green*) and the weighted summation (*dashed line*) of Cu(moAβ$_{4–9}$) (*red*) and Cu(GGH) (*blue*) basis spectra (Figure 2A, main text).

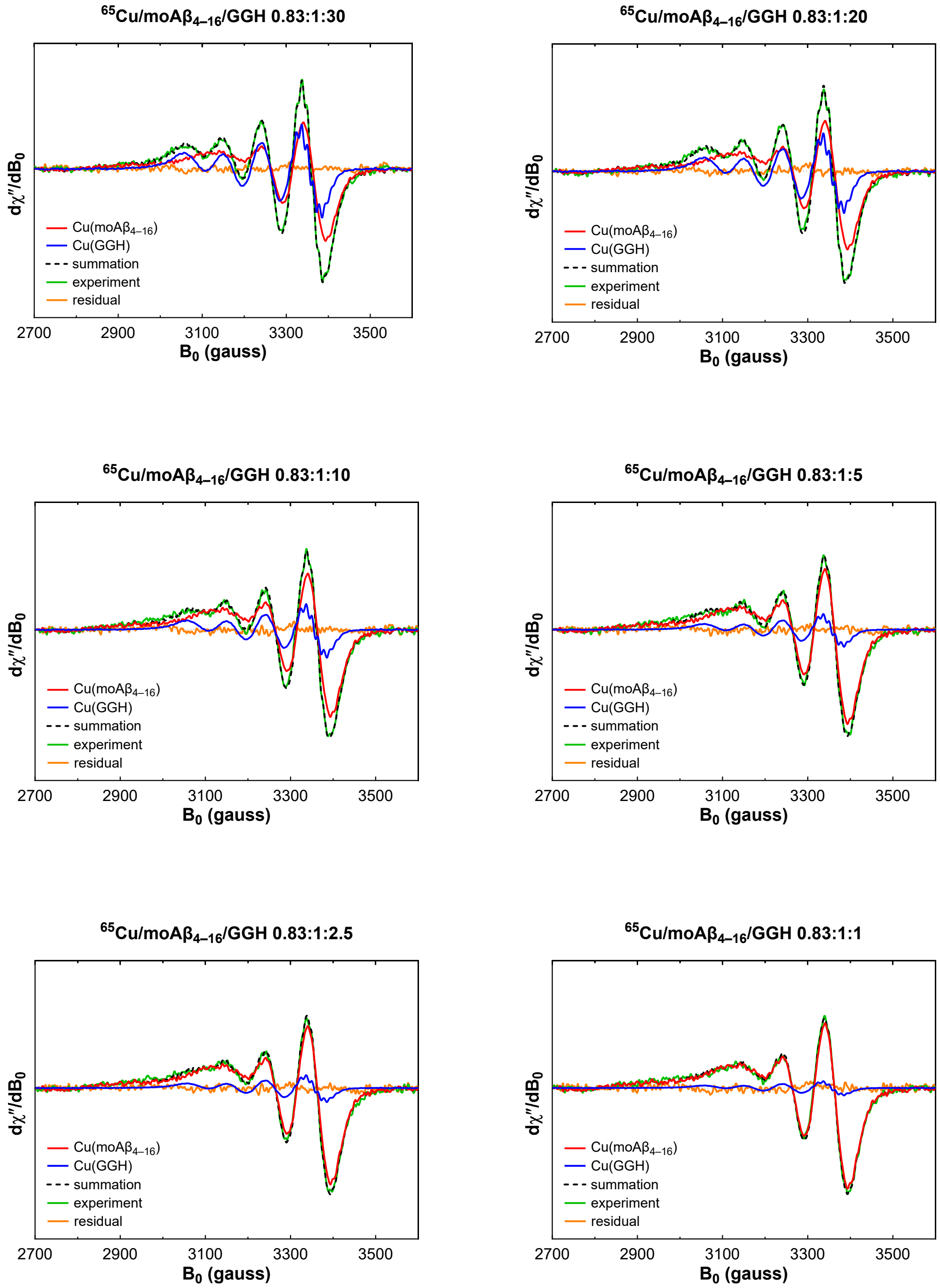

Figure S8. Decomposition of the room-temperature X-band (9.42 GHz) EPR spectra of Cu/moAβ$_{4–16}$/GGH mixtures prepared at molar ratios of 0.83 : 1 : *n*, where *n* = [GGH]$_T$ / [moAβ$_{4–16}$]$_T$ = 1–30. Percentages of each species are plotted in Figure 3B (main text) and were determined by minimising the difference (*orange*) between the experimental spectrum (*green*) and the weighted summation (*dashed line*) of Cu(moAβ$_{4–16}$) (*red*) and Cu(GGH) (*blue*) basis spectra (Figure 2A, main text).

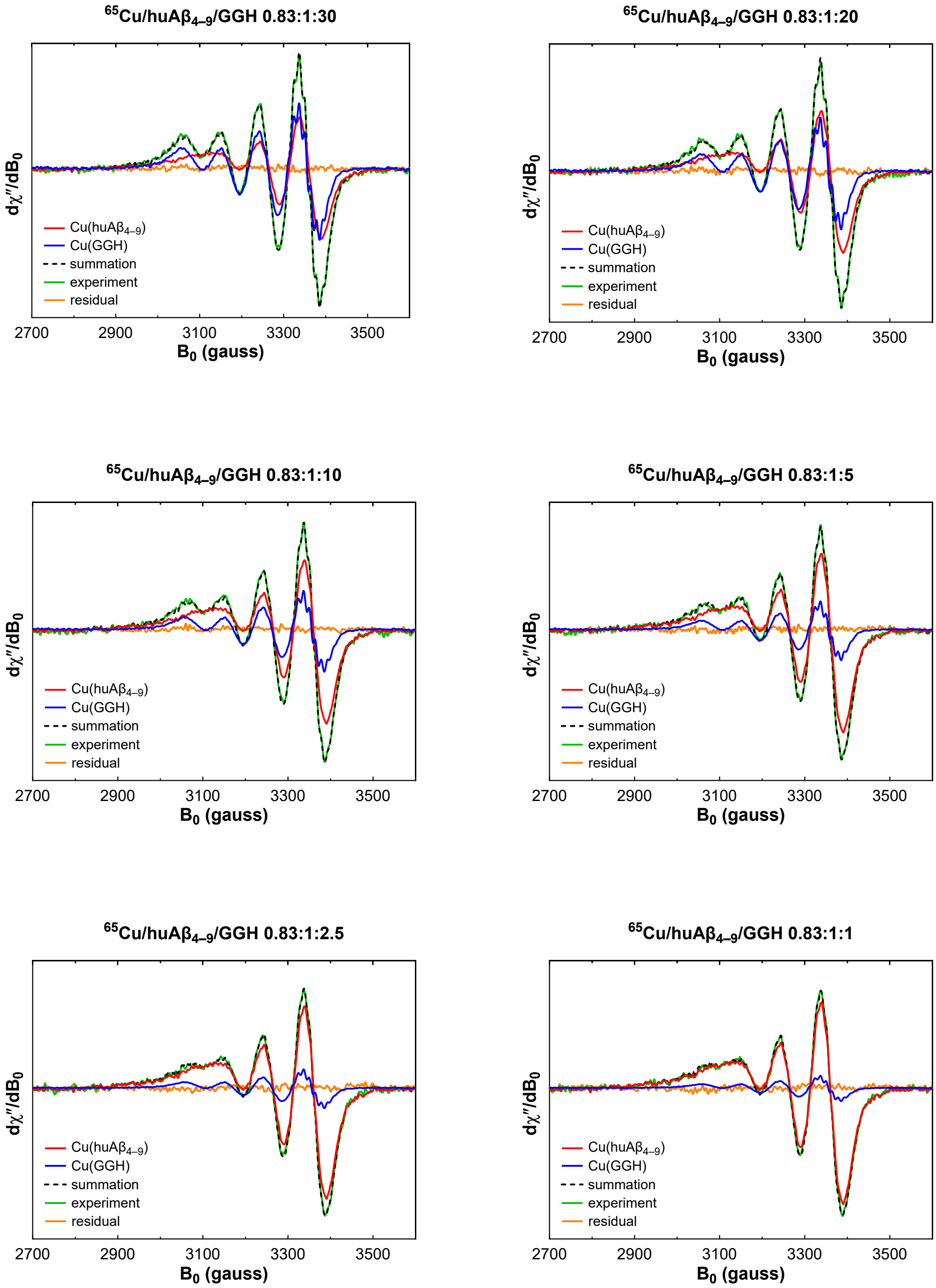

Figure S9. Decomposition of the room-temperature X-band (9.42 GHz) EPR spectra of Cu/huA$\beta_{4\text{–}9}$/GGH mixtures prepared at molar ratios of 0.83 : 1 : *n*, where $n = [\mathrm{GGH}]_\mathrm{T} / [\mathrm{huA}\beta_{4\text{–}9}]_\mathrm{T}$ = 1–30. Percentages of each species are plotted in Figure 3C (main text) and were determined by minimising the difference (*orange*) between the experimental spectrum (*green*) and the weighted summation (*dashed line*) of Cu(huA$\beta_{4\text{–}9}$) (*red*) and Cu(GGH) (*blue*) basis spectra (Figure 2A, main text).

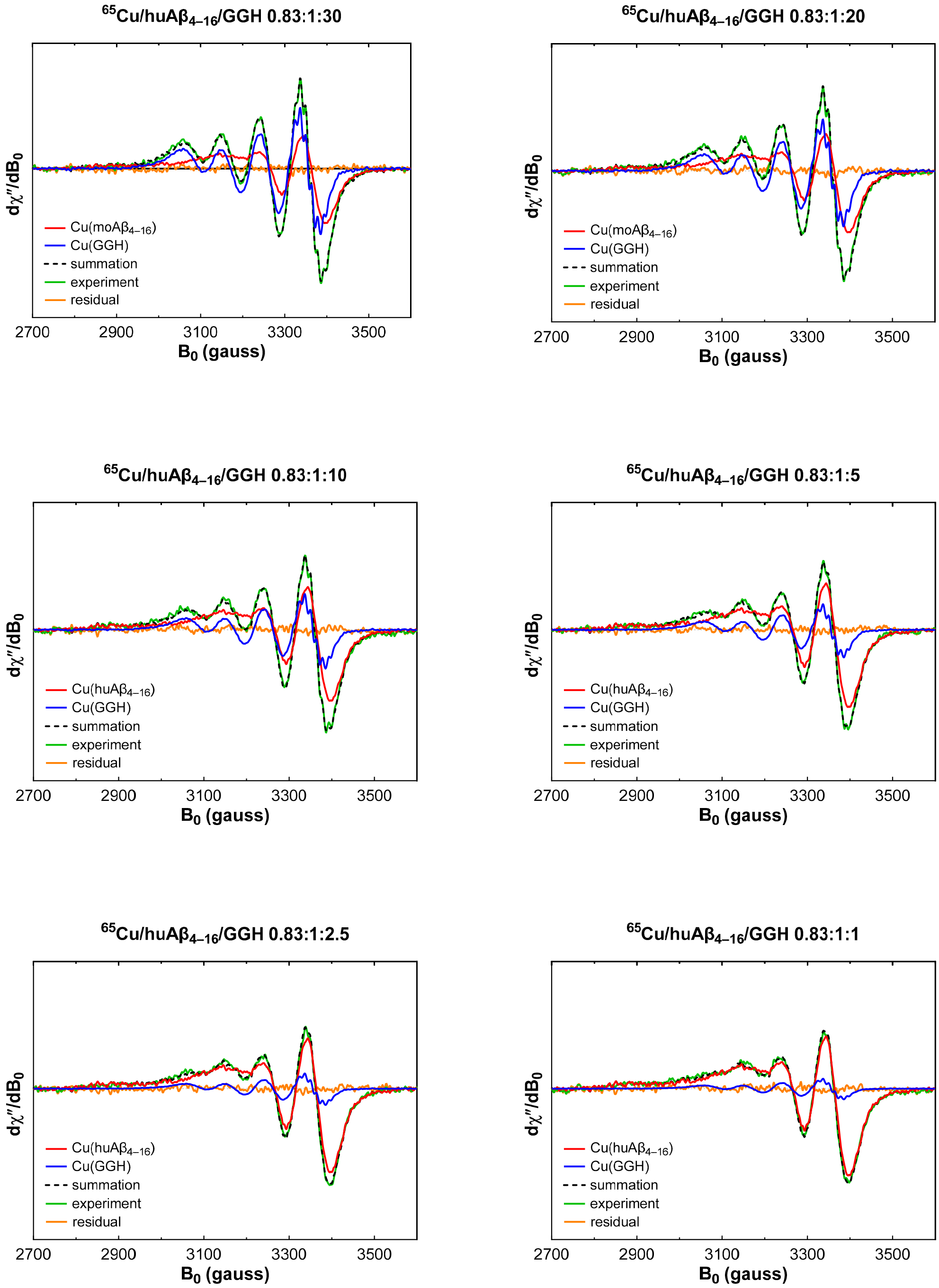

Figure S10. Decomposition of the room-temperature X-band (9.42 GHz) EPR spectra of Cu/huA$\beta_{4\text{–}16}$/GGH mixtures prepared at molar ratios of 0.83 : 1 : *n*, where $n = [\mathrm{GGH}]_\mathrm{T} / [\mathrm{huA}\beta_{4\text{–}16}]_\mathrm{T}$ = 1–30. Percentages of each species are plotted in Figure 3D (main text) and were determined by minimising the difference (*orange*) between the experimental spectrum (*green*) and the weighted summation (*dashed line*) of Cu(huA$\beta_{4\text{–}16}$) (*red*) and Cu(GGH) (*blue*) basis spectra (Figure 2A, main text).

Supplementary References

1. Bossak-Ahmad, K.; Frączyk, T.; Bal, W.; Drew, S.C. The sub-picomolar Cu^{2+} affinity of human serum albumin, *ChemBioChem* **2020**, *21*, 331–334. doi: 10.1002/cbic.201900435.

2. Peana, M.; Gumienna-Kontecka, E.; Piras, F.; Ostrowska, M.; Piasta, K.; Krzywoszynska, K.; Medici, S.; Zoroddu, M.A. Exploring the Specificity of Rationally Designed Peptides Reconstituted from the Cell-Free Extract of Deinococcus radiodurans toward Mn(II) and Cu(II). *Inorg. Chem.* **2020**, *59*, 4661–4684. doi: 10.1021/acs.inorgchem.9b03737.

3. Bossak-Ahmad K.; Mital, M.; Płonka, D.; Drew, S.C.; Bal, W. Oligopeptides Generated by Neprilysin Degradation of β-Amyloid Have the Highest Cu(II) Affinity in the Whole Aβ Family. *Inorg. Chem.* **2019**, *58*, 932–943. doi: 10.1021/acs.inorgchem.8b03051.

4. Mital, M.; Wezynfeld, N. E.; Frączyk, T.; Wiloch, M. Z.; Wawrzyniak, U. E.; Bonna, A.; Tumpach, C.; Barnham, K. J.; Haigh, C. L.; Bal, W.; Drew, S. C. A Functional Role for Aβ in Metal Homeostasis? N-Truncation and High-Affinity Copper Binding. *Angew. Chem. Int. Ed.* **2015**, *54*, 10460–10464. doi: 10.1002/anie.201502644.

5. Kotuniak, R.; Strampraad, M.J.F.; Bossak-Ahmad, K.; Wawrzyniak, U.E.; Ufnalska, I.; Hagedoorn, P.L.; Bal, W. Key Intermediate Species Reveal the Copper(II)-Exchange Pathway in Biorelevant ATCUN/NTS Complexes. *Angew. Chem.* **2020**, *59*, 11234–11239. doi: 10.1002/anie.202004264.

6. Sharrock, P.; Melník, M. Copper(II) acetates: from dimer to monomer. Can. J. Chem. **1985**, *63*, 52–56. doi: 10.1139/v85-009.

7. (a) Stoll, S.; Britt, R.D. General and efficient simulation of pulse EPR spectra. *Phys. Chem. Chem. Phys.* **2009**, *11*, 6614–6625. doi: 10.1039/b907277b; (b) Stoll, S.; Schweiger, A. EasySpin, a comprehensive software package for spectral simulation and analysis in EPR. *J. Magn. Reson.* **2006**, *178*, 42–55. doi: 10.1016/j.jmr.2005.08.013.
